# Valuing burden of premature mortality attributable to air pollution in major million-plus non-attainment cities of India

**DOI:** 10.1038/s41598-021-02232-z

**Published:** 2021-12-02

**Authors:** Moorthy Nair, Hemant Bherwani, Shahid Mirza, Saima Anjum, Rakesh Kumar

**Affiliations:** 1 Asian Development Research Institute (ADRI), Patna, BH India; 2 CSIR-National Environmental Engineering Research Institute (NEERI), Nagpur, MH India; 3 Academy of Scientific & Innovative Research (AcSIR), Ghaziabad, Uttar Pradesh India

**Keywords:** Environmental sciences, Environmental economics

## Abstract

Accelerating growth due to industrialization and urbanization has improved the Indian economy but simultaneously has deteriorated human health, environment, and ecosystem. In the present study, the associated health risk mortality (age > 25) and welfare loss for the year 2017 due to excess PM_2.5_ concentration in ambient air for 31 major million-plus non-attainment cities (NACs) in India is assessed. The cities for the assessment are prioritised based on population and are classified as ‘X’ (> 5 million population) and ‘Y’ (1–5 million population) class cities. Ground-level PM_2.5_ concentration retrieved from air quality monitoring stations for the NACs ranged from 33 to 194 µg/m^3^. Total PM_2.5_ attributable premature mortality cases estimated using global exposure mortality model was 80,447 [95% CI 70,094–89,581]. Ischemic health disease was the leading cause of death accounting for 47% of total mortality, followed by chronic obstructive pulmonary disease (COPD-17%), stroke (14.7%), lower respiratory infection (LRI-9.9%) and lung cancer (LC-1.9%). 9.3% of total mortality is due to other non-communicable diseases (NCD-others). 7.3–18.4% of total premature mortality for the NACs is attributed to excess PM_2.5_ exposure. The total economic loss of 90,185.6 [95% CI 88,016.4–92,411] million US$ (as of 2017) was assessed due to PM_2.5_ mortality using the value of statistical life approach. The highest mortality (economic burden) share of 61.3% (72.7%) and 30.1% (42.7%) was reported for ‘X’ class cities and North India zone respectively. Compared to the base year 2017, an improvement of 1.01% and 0.7% is observed in premature mortality and economic loss respectively for the year 2024 as a result of policy intervention through National Clean Air Action Programme. The improvement among 31 NACs was found inconsistent, which may be due to a uniform targeted policy, which neglects other socio-economic factors such as population, the standard of living, etc. The study highlights the need for these parameters to be incorporated in the action plans to bring in a tailored solution for each NACs for better applicability and improved results of the programme facilitating solutions for the complex problem of air pollution in India.

## Introduction

Air pollution has globally become a leading reason accounting for 22–53% of all deaths from cardiovascular diseases (CVD), ischemic heart diseases (IHD), stroke, chronic obstructive pulmonary disease (COPD) and lung cancer (LC)^1^. World Health Organisation ^86^ reported that India has the highest of total polluted cities and was the major contributor to annual particulate concentration at a global level. More than 90% of people in India breathe air that exceeds the World Health Organisation (WHO) interim target-1 (35 µg/m^3^)^2^. Balakrishnan et al.^63^ reported total mortality of 1.24 million in India due to air pollution (Ambient + Household) for the year 2017. The report claims that the estimated figure is an underestimation as additional diseases attributable to air pollution were unaccounted for. Welfare loss due to air pollution for the south Asian region, 2013 was reported to be 7.4% of the Gross Domestic Product (GDP)^3^. The welfare loss comprises of negative externalities due to ambient particulate, household particulate and ambient ozone pollution, whereas monetary loss due to other harmful pollutants such as black carbon, organic carbon, SO_2_, NO_2_, etc. was not considered. Pandey et al.^4^ estimated a total of 1.67 million premature mortality due to air pollution resulting in a total labour output productivity loss of 28.8 billion US$ for the year 2019 in India. Air pollution has almost topped the list of risk factors that cause mortalityin the country just below high blood pressure, tobacco and dietary risks. ^84^. Increased Urbanisation and industrialization activity together with unfavourable meteorological conditions^5–8^ are the prime reasons for increased health burden due to air pollution. Background concentration of India has always remained on the higher side, making the conditions eccentric to achieve the WHO safety limits.

Adverse health effects due to deteriorated air quality were of interest among researchers globally who then developed improved assessment methodologies with a focus to reduce the chances of larger uncertainties (^81–83^). Chowdhury and Dey et al.^9^ reported annual premature death of 8,11,000 cases in India due to mean PM_2.5_ exposure retrieved using satellite data from 2000 to 2010 by the Integrated Exposure Response (IER) model. The Non-Linear Power (NLP) used in the same study showed a lower estimation of 4,86,100 cases. The cases of premature mortality exceeded 1 million since 2012^10,11^ in the country. IER estimated cases were reported to have uncertainties due to limitations/assumptions such as (a) IER model does not account for all the non-accidental deaths thereby deflating the overall estimate of total potential cases, (b) Excess Relative Risk (RR) at higher PM_2.5_ concentration was predicted using alternative non-outdoor sources such as indoor heating/cooking, cigarette smoking assuming equal toxicity per dose across the outdoor sources; (c) IER includes information from additional sources such as active smoking, household heating and cooking which influences the hazard ratio estimate^12,13^. The global Exposure Mortality Model (GEMM) was developed by^12^ as an alternative to overcome these uncertainties. The model developed strictly relies on information related to outdoor PM_2.5_ particle exposure health risk and estimates 4.7 million excess deaths globally compared to that of Global Burden of Diseases (GBD) estimate for the year 2015^10^. Burnett et al.^12^ estimated total deaths using GEMM (Non-Communicable Diseases + Lower Respiratory Infection) and were reported to be more than twice in comparison with the IER model for India during 2015. Log-Linear (LL) functional form of Exposure Response Coefficient (ERC) is used globally to estimate excess health risk due to exposure to PM_10_ particles^14^. Whereas, GEMM is an extension of the LL model which includes other non-linear shapes as defined by the transformations^12^. The uncertainties associated with the previous GBD studies were addressed in the recently released GBD-2019 version^15^ which estimated total premature mortality of 0.98 million due to ambient air pollution for the year 2019 in India^4^.

Health risk cases owing to air pollution in Indian cities were estimated previously by several researchers^9,16,63,75,76^. But most of these studies were limited to the assessment of health endpoints without extending the scope towards monetary burden estimation. Monetary evaluation of health effects is beneficial to implement strategic decision-making through a robust policy framework and spread awareness against air pollution to minimise the overall loss due to air pollution externalities through improved quality of human health and well-being. There exist health-related monetary cost assessment studies for a few of the Indian cities such as Mumbai^17,18,77,78^, Delhi National Capital Region^19,78^, Agra ^79^, Chennai^80^, and Hyderabad^20^. Methods such as cost of illness, contingent valuation, hedonic wage, and benefit transfer methods are commonly employed to assess the economic loss attributable to air pollution^21^. Recently^4^, assessed a total forgone labour output due to air pollution (Ambient + Household + Ozone) for the year 2019 at India and state level using the labour output method.

**Figure 1:**
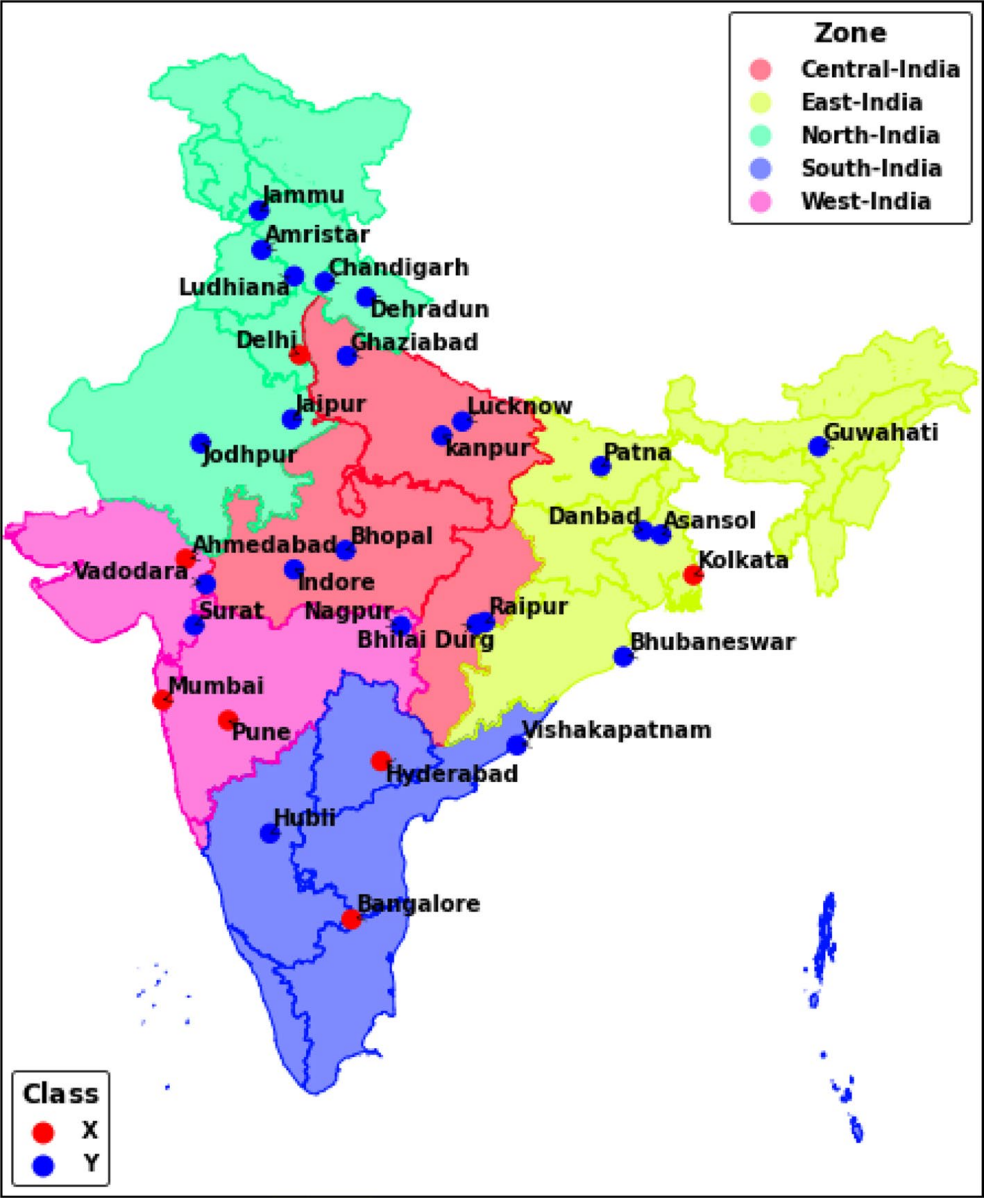
Study area showing 31 NACs with their respective class and zones. The map wasis generated using Python version 3.8.311.

As an initiative towards addressing the rising concern of air pollution in India, the Central Pollution Control Board, India (CPCB) has identified a total of 122 Non-Attainment Cities (NACs) across the country breaching the prescribed National Ambient Air Quality Standards (NAAQS) for particulate matter PM_10_ (Consecutively for 5 years from 2011 to 2015) and PM_2.5_ (2015 onwards). The cities are monitored and funded under National Clean Air Programme (NCAP) to ensure effective implementation of city-specific clean air action plan framework developed under this programme to achieve an overall targeted reduction of 20–30% in particulate matters by the year 2024^22^. India is one of the most populated countries in the world with varying levels of particulate matter concentration across its geography and needs a tailored micro-level policy intervention reinforced by scientific justifications to reverse the impact of air pollution. Thus, the study was formulated in a way to facilitate such intervention with an overall objective to (1) Estimate the monetary loss due to premature mortality attributed to air pollution (Excess PM_10_ & PM_2.5_) in major million-plus ‘X’ and ‘Y’ class NACs of India (Fig. 1) for the year 2017. The government of India has classified cities based on the population of the urban agglomeration area of the city into classes ‘X’ (population > 5 million), ‘Y’ (5 million > population > 0.5 million) and ‘Z’ (population < 0.5 million). (2) Potential health benefits in monetary terms under the National Clean Air Programme (NCAP) pollution control regime through scenario setting of targeted reductions. To reduce the complexity of the study by including all the NACs, it was decided to consider all ‘X’ class NACs with at least one major ‘Y’ class NACs having the highest population for all the states of India and were chosen based on the Non-attainment list published by Central Pollution Control Board (https://cpcb.nic.in/uploads/Non-Attainment_Cities.pdf) and Sixth Central Pay Commission's city classification (https://doe.gov.in/sites/default/files/21-07-2015.pdf).

## Methodology

**Figure 2:**
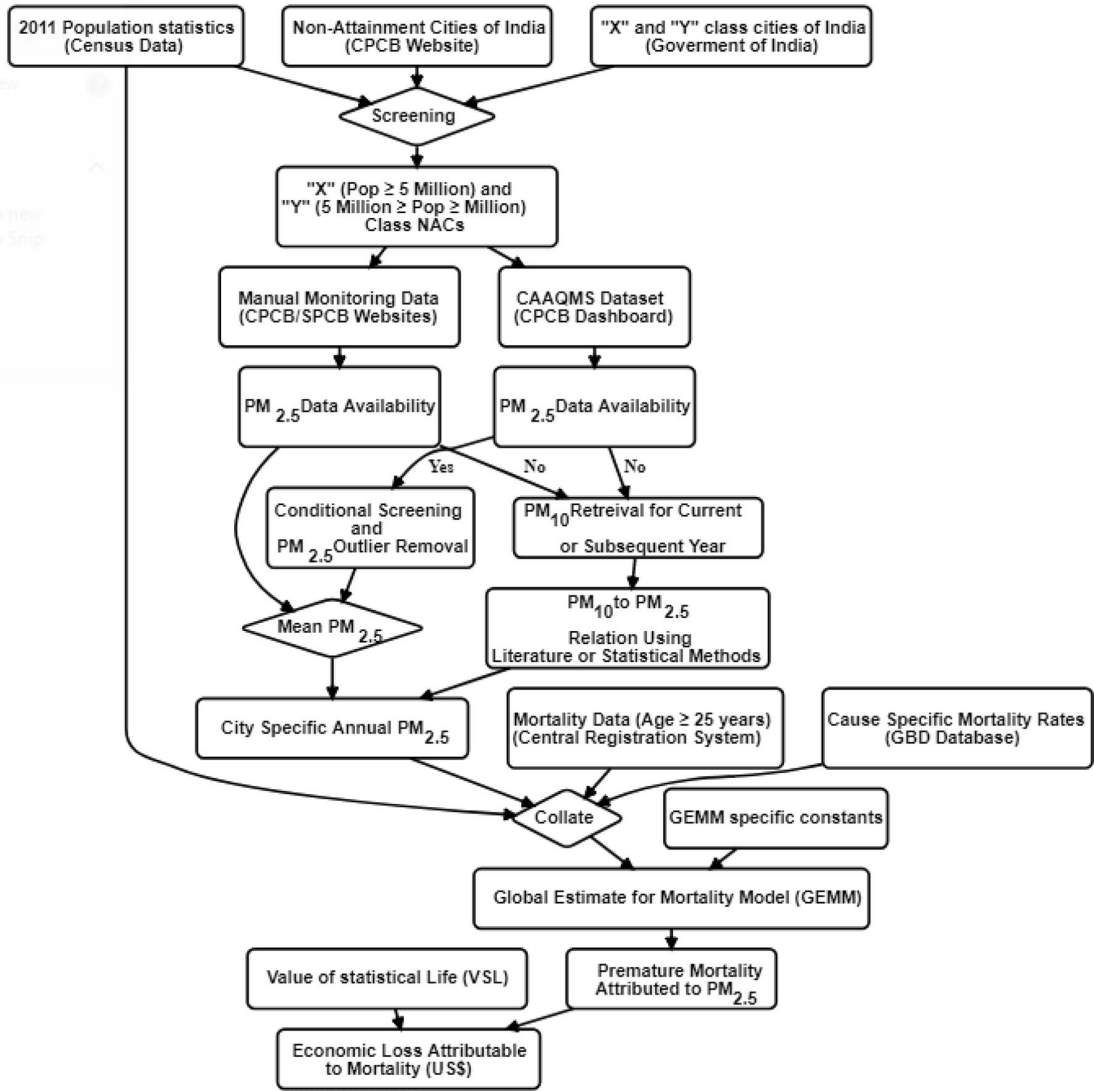
Generic flowchart for damage cost assessment due to health risk. Abbrevations mentioned includes CPCB: Central Pollution Control Board; SPCB: State Pollution Control Board; GBD is Global Burden of Diseases; GEMM is Global Estimate for Mortality Model. The graph was generated using R software version 4.0.5.Generic flowchart for damage cost assessment due to health risk. Abbrevations mentioned includes CPCB: Central Pollution Control Board; SPCB: State Pollution Control Board; GBD is Global Burden of Diseases; COPD: Chronic Obstructive Pulmonary Disease; LRI: Lower Respiratory Infection; LC: Lung Cancer; IHD: Ischemic Heart Disease; NCD: Non-Communicable Diseases.

Generic data flow to assess economic losses due to cause specific PM_2.5_ mortality (age > 25) is shown in Fig. 2. The comprehensive methodology followed in retrieving each of the datasets is explained in further sections.

### Ground level PM_2.5_monitoring

PM_2.5_ is said to be the better predictor for health risk compared to PM_10_^23,24,85^. Annual PM_2.5_concentration data was retrieved for the year 2017 from the Central Pollution Control Board (CPCB) and respective State Pollution Control Board (SPCB) websites (Detailed in Supplementary Table S2). Consistency in data retrieval was maintained by considering only those stations which have data available for more than 104 days^25^ but was made limited only for continuous monitoring stations and not manual stations in the current study due to the limited availability in manual monitored data. The retrieved data was manually screened by eliminating daily averaged concentrations deviating away from 2 to 1000 µg/m^3^ range (Saini & Sharma 2019). Linear correlation developed between PM_10_-PM_2.5_ was used to account for the missing PM_2.5_concentrations from PM_10_. It was observed that PM_10_-PM_2.5_ relation couldn’t be just developed using 2017 data for certain stations due to large data gaps, hence data from 2018 was considered for developing the correlation with an assumption that clean air action plan for all the NACs being initiated with the base year 2019[^22^] and had no substantial improvement in air quality for the immediately previous year. Thus developed correlation values were utilised to predict the respective PM_2.5_ for the year 2017. Due to the lack of either PM_10_ or PM_2.5_ monitoring by the government-operated instruments in cities like Kanpur, Dhanbad, Patna and Dehradun have necessitated for direct conversion of PM_10_ to PM_2.5_ using the ratio factor analysed from literature^26–30^ for developing the relationship.

### Cause-specific premature mortality assessment attributable to PM_2.5_

The global Exposure Mortality Model (GEMM) proposed by Burnet et al.^12^ was employed to estimate the Relative Risk (RR) of premature mortality due to ambient PM_2.5_ exposure. The model has overcome 2 major limitations of the IER model and is well highlighted by Maji^31^. The model considers all non-accidental mortality due to Non-Communicable Disease (NCD) and Lower Respiratory Infection (LRI) attributable to ambient PM_2.5_ exposure based on previous cohort’s studies. Relative Risk (RR) and Mortality (age > 25 years) due to GEMM (NCD + LRI) along with individual cause-specific cases such as Chronic Obstructive Pulmonary Disease (COPD), Ischemic Heart Disease (IHD), Stroke, Lung Cancer (LC) and LRI were estimated using Eq. (1) ^12^ and Eq. (2) ^31–33^ respectively.

1 $$ RR = \exp [\theta \log (z/\alpha + 1)/1 + \exp \{ - (z - \mu )/v\} ] $$

where ‘*θ*’, ‘*α*’, ‘*μ*’, ‘*ν*’, are constants specific to age and diseases (*i*) in the GEMM. These constants are provided by Burnet et al.^12^ in his supplementary information and are based on 15 cohorts having non-linear PM_2.5_-mortality association. Since the India specific cohorts are very limited and are still in the phase of expansion, Asian cohorts from China and Tapei has been included in GEMM as complementary. ‘*z*’ is excess PM_2.5_ concentration above the threshold to which the population is exposed. The threshold PM_2.5_ concentration below which there are no substantial negative health effects mentioned in his study was 2.4 µg/m^3^.

2 $$ \Delta E_{Mortality = } \left[ {{\raise0.7ex\hbox{${\left( {RR - 1} \right)}$} \!\mathord{\left/ {\vphantom {{\left( {RR - 1} \right)} {RR}}}\right.\kern-\nulldelimiterspace} \!\lower0.7ex\hbox{${RR}$}}} \right] \times B_{i } $$

‘*RR*’ estimated using Eq. (1) was incorporated in Eq. (2) to estimate the excess mortality attributable to PM_2.5_ ($\Delta E_{Mortality }$). ‘$\left( {RR - 1} \right)/RR$’ is attributable fractions defined as the proportion of mortality disease burden among exposed populations attributable to risk factors ^34^. $B_{i }$ is the baseline mortality for the year 2017 specific to individual diseases $\left(i\right)$ and city for age > 25 years. All-cause baseline mortality (age > 25) for each city was adjusted from percentage share of age-wise death rates and total mortality retrieved at the state and district urban level respectively from the Civil Registration System (CRS) India^35^ using the population distribution data at the state, district and city levels being projected to the study period (https://censusindia.gov.in/). The percentage shares of age-wise death rates were assumed to be the same for both state and district urban regions. The percentage shares of cause-specific mortality (age > 25) for individual states were gathered from the Global Burden of Disease (GBD) India Compare Data Visualization interface^36^. The percentage share from GBD was then directly incorporated into city shares to estimate cause-specific PM_2.5_ mortality cases (age > 25).

### Economic losses due to PM_2.5_ mortality

Due to the unavailability of market value for human lives^37^, the monetary burden due to health risk was calculated using the method of Value of Statistical Life (VSL). VSL is an individual’s willingness to pay (WTP) to avoid the risk of mortality^38,39^. The 2 most common methods to estimate WTP are contingent valuation (CV) using a questionnaire^40–42^ and Compensating Wage Differential (CWD) using the Hedonic wage function approach^43^. The hedonic wage approach is widely used by various researchers^37,44–48^ in estimating India specific VSL. Due to the unavailability of the city or state-specific VSL values corresponding to 31 NACs, the study estimated the economic cost due to mortality at the state level by benefit transfer method using Eq. (3) ^49^ considering VSL of India as the base.

3 $$ EC_{k,2017} = EC_{Ind,year} \times \left[ {\frac{{G_{k,2017} }}{{G_{Ind,year} }}} \right]^{\varepsilon } \times \left[ {1 + \% \Delta G + \% \Delta CPI} \right]^{\beta } \times PPP_{2017} $$

where ‘$$EC_{k,2017}$$’ US$ is the economic cost of mortality in the respective state ‘$$k$$’ of the NACs for the year 2017. The complete list of NACs and their belonging states were mentioned in Supplementary material Table S1. ‘$$EC_{Ind,year}$$’ (₹) is the mortality cost in India for a known year. Shanmugan et al.^50^ carried out a VSL study in India during the year 1996–1997 and the value ranged from ₹ 14 million to ₹ 19 million. Further^48^, estimated VSL for India in the range of ₹ 6.4 million to ₹ 15 million. In the current study, the most recent value ie., ₹44.69 million estimated in the year 2016^46^ was used. ‘$$G_{k,2017}$$’ and ‘$$G_{Ind,year}$$’ is the GDP per capita (₹) for the state ‘$$k$$’ ‘K’ -2017 and India-2016 respectively and was retrieved from (https://www.india.gov.in/website-directorate-economics-and-statistics). ‘$$\% \Delta G$$’ and ‘$$\% \Delta CPI$$’ are the percentage change in GDP per capita and Consumer Price Index (CPI) of the state ‘$$k$$’ respectively for the year 2017 from 2016. ‘$$CPI$$’ data was retrieved from (https://data.gov.in/). Purchasing Power Parity (PPP) exchange rates was retrieved (https://data.worldbank.org/indicator/PA.NUS.FCRF?locations=IN) for the year 2017 was used to convert Indian Nation Rupee (INR₹) to US Dollar (US$). ‘$$\varepsilon $$’ is the elastic coefficient of WTP and is considered 1.0^51,52^. ‘$$\beta$$’ is the income elasticity and is recommended to be 0.8^53,54^.

4 $$ PM_{2.5} Mortality\, Economic \,loss = EC_{k,2017} \times MC_{i} $$

Total economic loss (US$) due to PM_2.5_ mortality for state ‘$$k$$’ of NACs were calculated using Eq. (4). ‘$$MC_{i} $$’ is disease (‘*i*’) specific mortality cases.

### Scenario modelling on policy intervention

NCAP was released by the Government of India with an overall national target of 20–30% reduction in PM by 2024 keeping 2017 as the base year. Scenario modelling for the suggested target was attempted using Eq. (1)–(4) to estimate the improvement in terms of monetary benefit (US$) with an optimum reduction in PM_2.5_ by 30%. The baseline incidence of disease-specific mortality was assumed to be the same as that of 2017. The urban population (age > 25) statistics for the year 2024 provided by (https://censusindia.gov.in/) was used in the assessment.

## Results and discussion

### Annual average of PM_2.5_ concentration

Data availability at continuous and manual monitoring stations for each NACs was shown in Supplementary Table S2. Missing data/data gaps of the NACs were completed using linear regression relation developed between the retrieved PM_2.5_ and PM_10_ data with previous studies as references. The detailed linear regression models and equation developed/retrieved were shown in Supplementary Fig. S1 and Table S3 respectively. The Pearson’s correlation coefficient (r) ranged from 0.65 to 0.97 showing a strong linear correlation between the two pollutants of size 2.5 and 10 µm.

**Figure 3:**
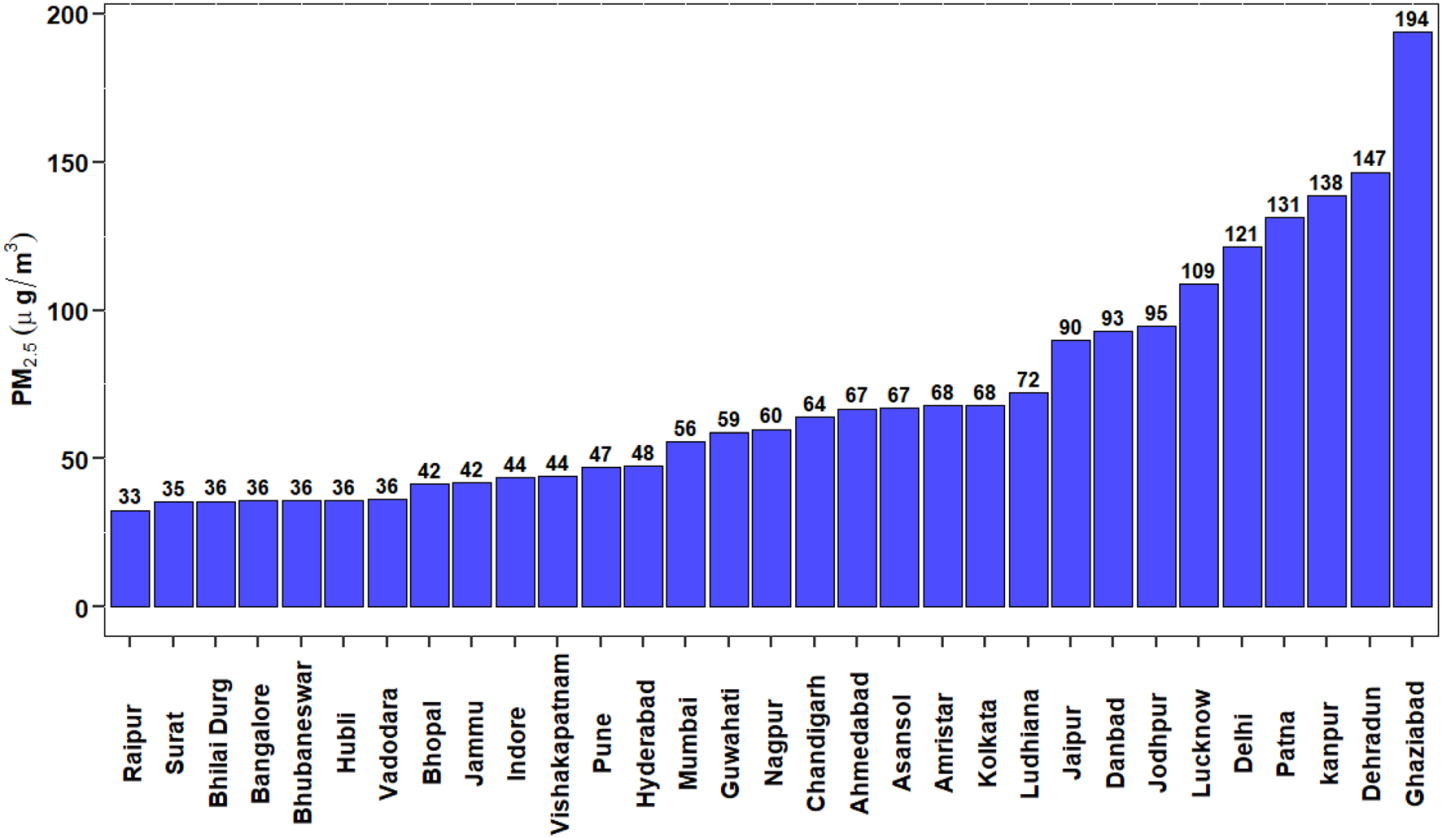
Annual average of PM_2.5_ concentration (µg/m^3^) for 31 NACs for the year 2017. The graph is was generated using R software version 4.0.5.

Figure 3 shows the annual PM_2.5_ average for 31 NACs for the year 2017. PM_2.5_ concentration ranged from 33 to 194 µg/m^3^. The maximum concentration was found to be approximately 5 times the NAAQS-India prescribed annual average (ie., 40 µg/m^3^)^25^. The highest concentration was observed in cities such as Delhi (121 µg/m^3^), Dehradun (147 µg/m^3^), Ghaziabad (194 µg/m^3^), Kanpur (138 µg/m^3^), Lucknow (109 µg/m^3^) and Patna (131 µg/m^3^). These cities being located in Indo-Gangetic Plain (IGP) region are subjected to higher pollution loading due to their indigenous pollution sources along with various other exacerbating factors such as poor metrological conditions^55^, topography, atmospheric dynamics changes^56^ and cross-boundary movement of pollutants from the neighbouring state and outside India origin (David et al., 2019)^57–59^. Other IGP cities such as Chandigarh, Amritsar, Ludhiana, Kolkata and Asansol also have exhibited high pollution levels (> 60 µg/m^3^). The lowest concentration (< 40 µg/m^3^) was observed in the cities belonging to the state of Karnataka, Gujarat, Chattisgarh and Odisha because of the low population density and favourable meteorological conditions in the regions.

### Health risk assessment

The total mortality from IHD, Stroke, COPD, and LC is categorised under NCD^60^. Further, these causes along with LRI are represented as GEMM 5-COD (Cause of Death) in the study. To eliminate double-counting, mortality due to GEMM 5-COD was segregated from GEMM (NCD + LRI) and are reported as NCD-other.

#### PM_2.5_—All-cause mortality

**Figure 4:**
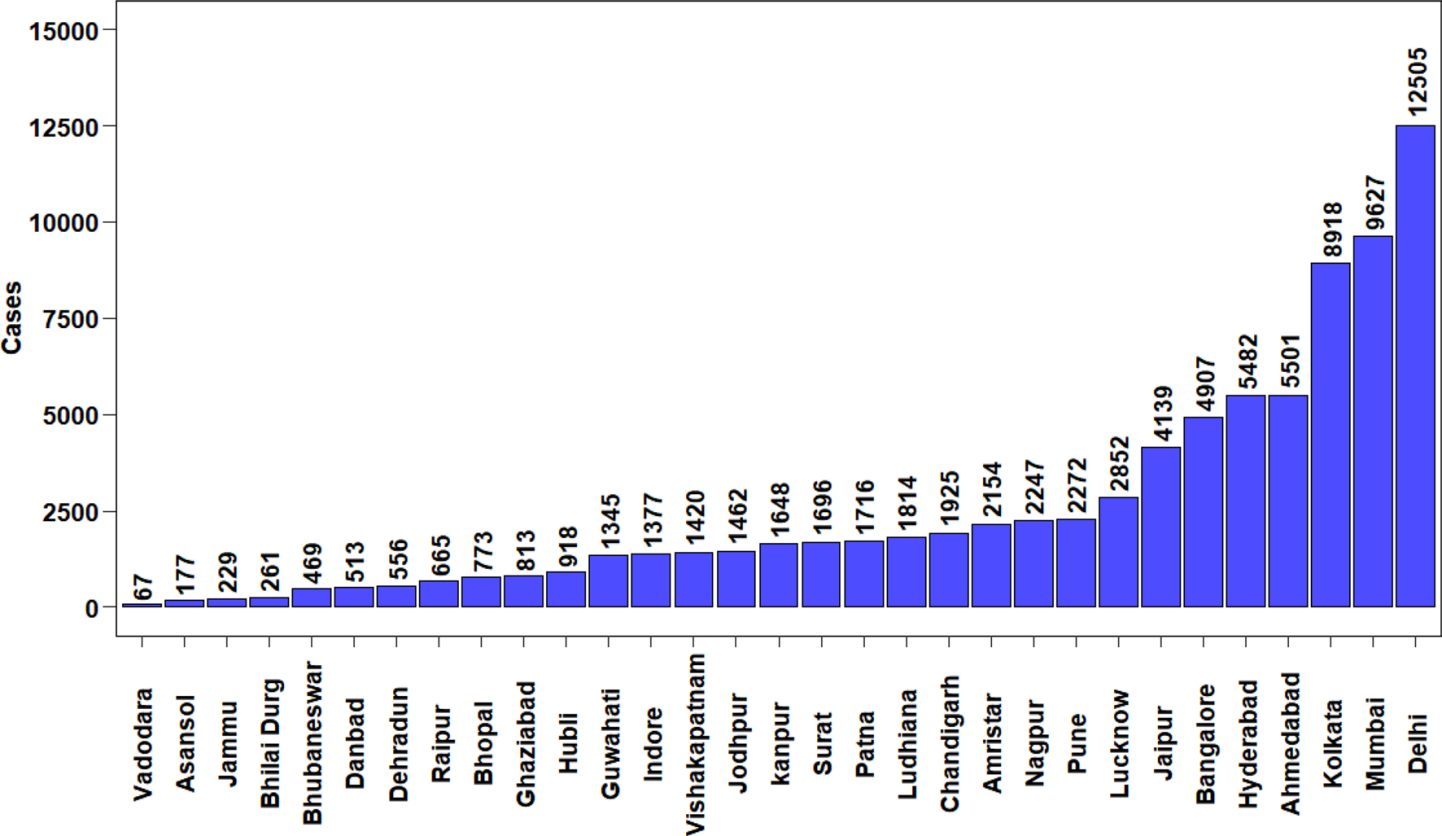
Estimated PM_2.5_ all cause mortality cases for the year 2017. The graph is was generated using R software version 4.0.5.

Estimated PM_2.5_ all-cause mortality cases for the year 2017 is shown in Fig. 4. A total of 80,447 ^30^ mortality cases GEMM (NCD + LRI) was estimated for 31 NACs for the year 2017. Out of the total all-cause PM_2.5_ mortality cases estimated, 61.2% and 38.8% are from seven ‘X’ and twenty-four ‘Y’ class cities respectively. Highest mortality case was observed for Delhi- 12,505 [95% CI 11,094–13,872] followed by other ‘X’ class cities such as Mumbai- 9627 [95% CI 8494–10,736], Kolkata- 8918 [95% CI 7880–9932], Ahmedabad- 5501 [95% CI 4860–6127], Hyderabad- 5482 [95% CI 4832–6120] and Bangalore – 4907 [95% CI 4319–5487]. In the case of ‘Y’ class cities, total mortality cases ranged from 4138 [95% CI 3664–4600] for Jaipur to 66 [95% CI 59–75] for Vadodara. Despite observing relatively low PM_2.5_ concentration in ‘X’ class cities like Bangalore (36 µg/m^3^), Hyderabad (48 µg/m^3^), Ahmedabad (67 µg/m^3^) and Kolkata (68 µg/m^3^) mortality cases were found high in comparison to those cities which had higher PM_2.5_ concentration. This is due to the difference in baseline incidence cases and exposed population of those cities (Details in Supplementary Sheet: Table S4, Table S5 and Fig. S2). Similar observations were also reported for a study in China^49^. Burnet et al.^12^ reported total mortality for India using GEMM ie., NCD + LRI for the year 2015 to be 2219 thousand. Recently a study carried out by Maji^31^ for china reported a total mortality GEMM (NCD + LRI) of 1930 thousand for the year 2017 and then a decreases by 9% for the year 2019. David et al.^11^ showed that 49% of total mortality (Estimated using the IER method) for the year 2012 is attributed to the IGP region. In the current study, cities situated in the IGP region like Chandigarh, Amritsar, Ludhiana, Delhi, Ghaziabad, Kanpur, Lucknow, Patna, Kolkata and Asansol together has accounted for 43% of the GEMM (NCD + LRI) mortality. Table 1 shows the overall zonal statistics of the study results. North, Central, East, West and South India zone constitute 30.1%, 11.1%, 16.3%, 26.6% and 15.8% respectively to the overall PM_2.5_ mortality.

**Table 1:** Zonal statistics summarised from the study.

Sl. No	Description	North-India	Central-India	East-India	West-India	South-India
1	Total Number of NAC	7 [X class: 1; Y class: 6]	8 [X class: 0; Y class: 8]	6 [X class: 1; Y class: 5]	6 [X class: 3; Y class: 3]	4 [X class: 2; Y class: 2]
2*	PM_2.5_ (µg/m^3^) Concentration	78.7 [41.7–121.3]	92.6 [32.6–193.7]	70.7 [35.8–102,131.14]	50.1 [35.3–66.7]	40.8 [35.7–47.6]
3	PM_2.5_ – total premature mortality cases	24,227 [95% CI 21,461–26,914]	8945 [95% CI 7917–9945]	1213,977 138 [95% CI 11,468,613–14,626450]	21,409 [95% CI 18,891–23,875]	12,727 [95% CI 11,210–14,219]
4*	Percentage share of all cause mortality attributed to PM_2.5_exposure	14.1% [10.1% to 18.0%]	12.9% [7.6% to 18.4%]	12.57% [7.3% to 1416.62%]	10.8% [8.5% to 12.8%]	9.3% [8.8% to 9.9%]
5	Economic Damage (Million US$)for the year 2017	38,511 [95% CI 39,677–37,381–39,677]	4420 [95% CI 4539–4304–4539]	7,138 179 [95% CI 7306–6,9737,012–7350]	24,938 [95% CI 25,455–24,432–25,455]	15,135 [95% CI 15,389–14,886–15,389]
6*	Percentage change based on target scenario	+ 0.03% [− 5.9% to + 5.4%]	+ 4.6% [− 4.3% to + 14.6%]	- + 0.61% [− 6.5% to + 5.39.2%]	− 4.1% [− 12.4% to + 0.5%]	− 2.3% [− 3.9% to − 0.1%]

( +) indicates increase.

(−) Indicates decrease.

*Average [Min – Max] is the format followed.

#### PM_2.5_—Cause specific mortality

GEMM 5-COD for total of 31 NACs was estimated to be 72,903 [95% CI 62,514–82,626]. GEMM 5-COD was found highest for Delhi at 10,221 [95% CI 9069–11,280] followed by other ‘X’ class cities such as Mumbai at 9214 [95% CI 7885–10,466], Kolkata at 8663 [95% CI 7252–9981], Ahmedabad at 5265 [95% CI 4560–5926], Bangalore at 4437 [95% CI 3818–5031] and Hyderabad at 5250 [95% CI 4544–5920]. Chen et al.^61^ estimated premature mortality $(\sum \left[ {{\text{Stroke}},{\text{ IHD}},{\text{ LC}},{\text{ LRI}},{\text{COPD}}} \right]:{ }5 - {\text{COD}})$ using IER for Delhi, Mumbai and Hyderabad to be 10,200, 9500 and 5200 respectively for the year 2016. In his study, PM_2.5_ values were retrieved only from Beta attenuation monitors and not manual monitoring stations which were prevalent during the study period. This must have accounted for limited spatial coverage of data showing a moderate variation in PM value towards the higher side compared to our study and thus capturing a higher number of cases for all 3 cities. Similarly, a study carried out by Maji et al.^51^ for Mumbai and Delhi reported total premature mortality (5-COD) due to PM_2.5_ for the year 2015 to be 13,196 and 14,844 respectively based on the IER method. The reported estimates were found higher than the current study for two reasons (a) High PM_2.5_ value; (b) Assumption of India-level constant baseline mortality rate for both ‘X’ class cities. Total mortality reported at the country level considers mortality due to both urban and rural styles of living^60,62^. ^63^ reported that 38.8% of total air pollution mortality in the country is attributable to household air pollution using filthy cooking fuel. The same study also reported that the figure doesn’t hold true for ‘X’ class cities like Delhi whose percentage share towards household air pollution is approximately 0.4%. Hence considering the country level baseline incidence at the city level may result in overestimated premature mortality cases. Maji et al.^16^ carried a similar study for megacities in India using IER and reported total premature mortality (5-COD) to be 14.8, 10.5, 7.3, 4.8 thousand for Delhi (132 µg/m^3^), Mumbai (81 µg/m^3^), Kolkata (79 µg/m^3^) and Bangalore (65 µg/m^3^) respectively. High mortality reported in his study was attributable to higher excess PM_2.5_ concentration compared to the present study. Pune among ‘X’ class city showed the lowest at 2188 [95% CI 1871–2490]. Jaipur among ‘Y’ class cities showed the highest GEMM 5-COD at 3588 [95% CI 2972–4155] followed by other cities like Lucknow at 2310 [95% CI 1938–2650], Amritsar at 2154 [95% CI 1930–2634], Nagpur at 2143 [95% CI 1835–2432], Ludhiana at 1806 [95% CI 1620–1981], Chandigarhat 1727 [95% CI 1469–1968], Surat at 1663 [95% CI 1434–1882], Patna at 1418 [95% CI 1213–1606], Vishakhapatnam at 1360 [95% CI 1173–1538], Kanpur at 1322 [95% CI 1113–1512], Jodhpur at 1263 [95% CI 1047–1462], Indore at 1250 [95% CI 1046–1443], Guwahati at 1003 [95% CI 810–1184], Hubli at 830 [95% CI 715–942], Bhopal at 702 [95% CI 587–811], Ghaziabad at 650 [95% CI 549–741], Raipur at 562 [95% CI 460–659], Dehradun at 468 [95% CI 397–532], Dhanbad at 412 [95% CI 352–467], Bhubaneswar at 351 [95% CI 283–415], Jammu at 224 [95% CI 190–256], Bhilai Durg at 222 [95% CI 182–260], Asansol at 172 [95% CI 144–199] and Vadodara at 65 [95% CI 56–74]. North, Central, East, West and South India zone constitute 28.8%, 10.2%, 16.5%, 28.2% and 16.3% respectively to the total GEMM 5-COD (Table 1).

**Figure 5:**
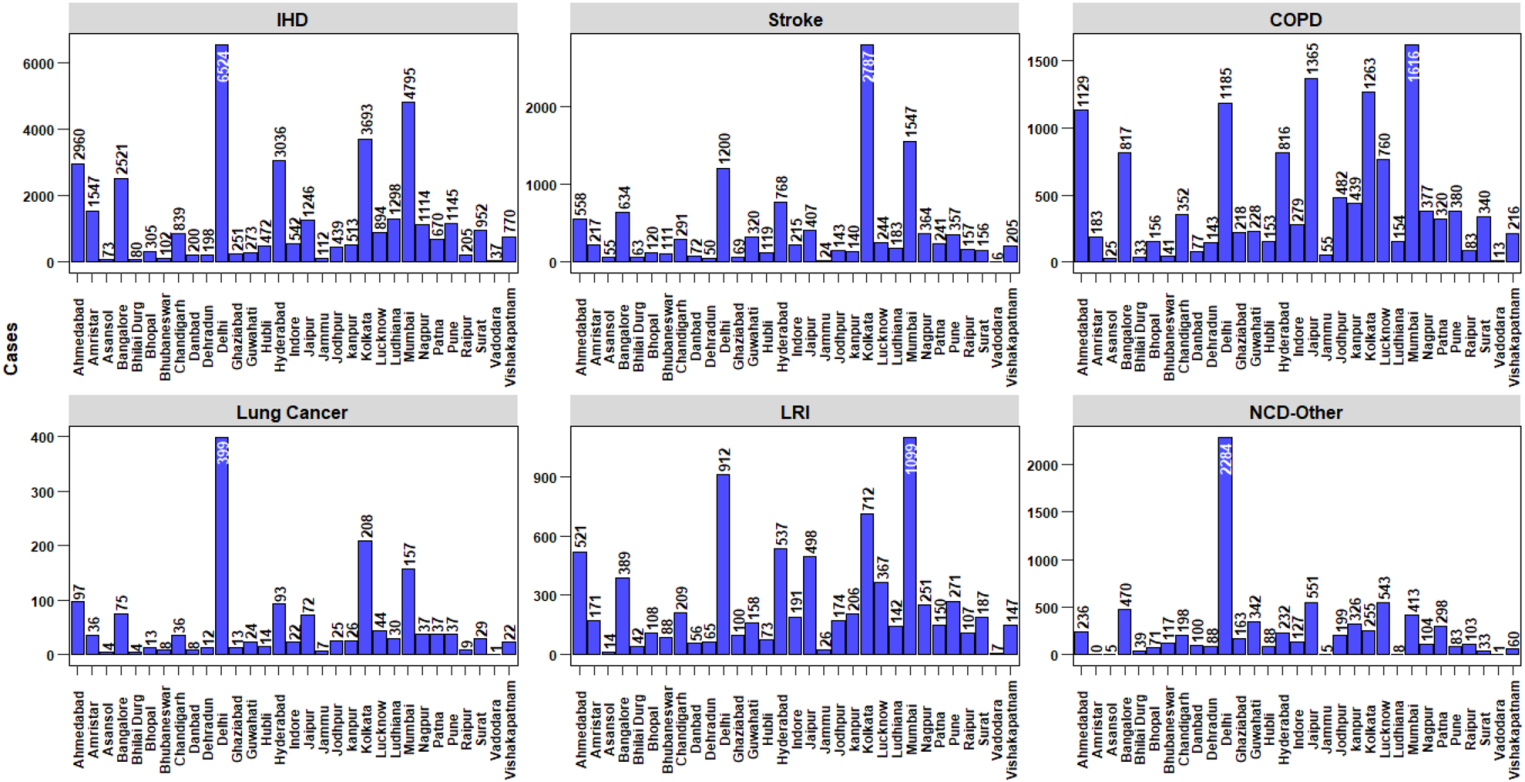
Estimated PM_2.5_ cause specific mortality cases. The graphs is were generated using R software version 4.0.5.

Figure 5 shows the PM_2.5_ cause specific mortality estimated for 31 NACs. Highest cause specific mortality cases toggled within the ‘X’ class cities such as Delhi, Mumbai and Kolkata. Highest total mortality cases with 37,805 [95% CI 35,899–39,670] was attributable to IHD followed by COPD – 13,698 [95% CI 10,317– 16,874], Stroke – 11,823 [95% CI 8767–14,674], LRI- 7979 [95% CI 6229–9529], NCD-Other-7544 and LC-1599[95% CI 1302–1879]. Delhi showed Highest mortality at 6524 [95% CI 6217–6822], 399 [95% CI 465–327] for IHD and LC respectively. Kolkata at 2787 [95% CI 2069–3454] for stroke and Mumbai at 1616 [95% CI 1212–1999] and 1099 [95% CI 855–1318] for COPD and LRI respectively. Jaipur being a ‘Y’ class city showed higher mortality cases for most of the causes. In case of COPD, Jaipur 1365 [95% CI 1033–1673] was second highest among the 31 NACs. Amritsar showed highest IHD mortality among ‘Y’ class cities at 1547 [95% CI 1469–1623]. Lowest mortality was estimated for vadodara at 37 [95% CI 35–39], 6 [95% CI 5–8], 13 [95% CI 10–17], 1 [95% CI 1–1], 7 [95% CI 6–9] for IHD,Stroke, COPD, LC and LRI respectively.

**Figure 6:**
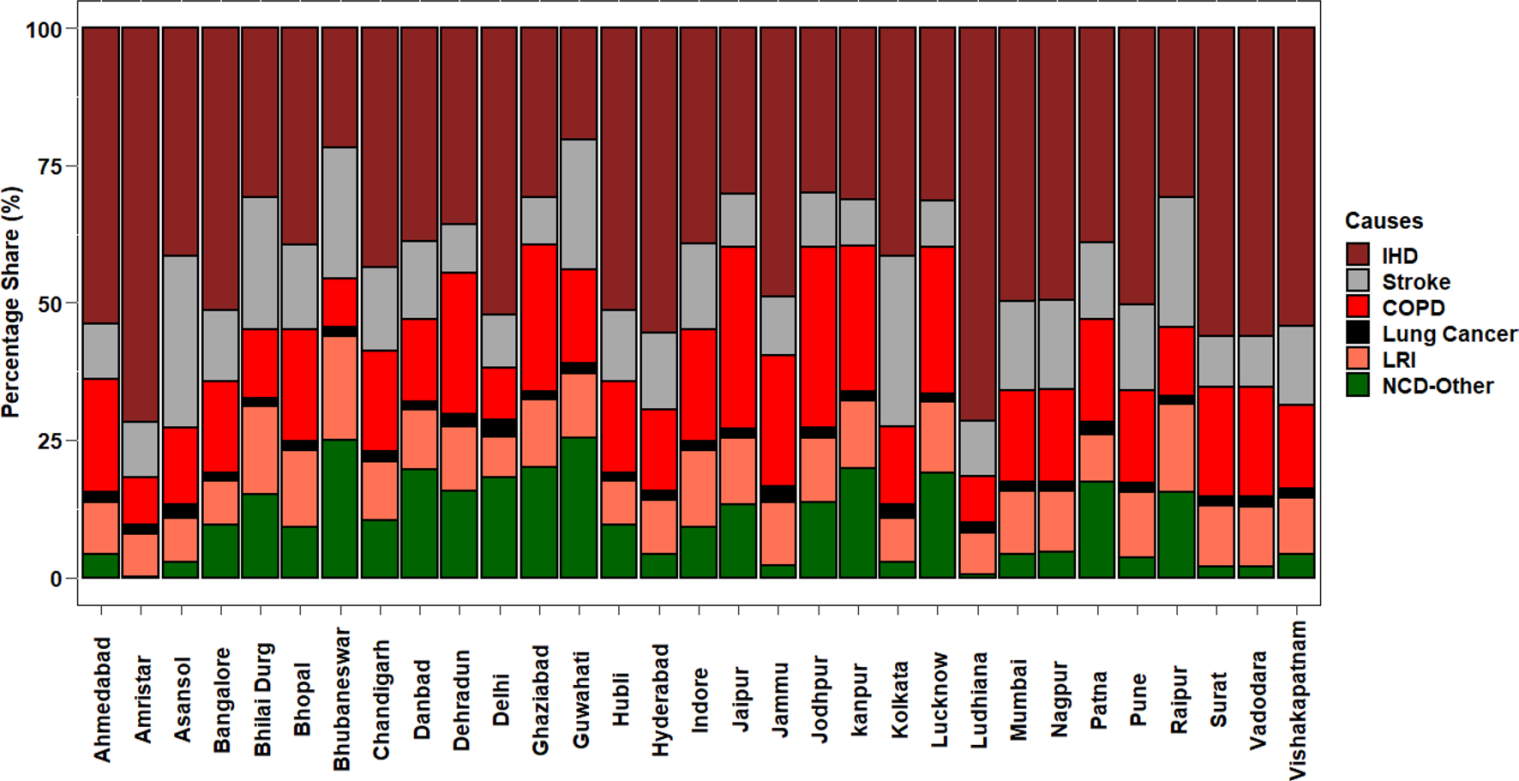
Estimated PM_2.5_ cause specific mortality shares. The graph wasis generated using R software version 4.0.5 The graph is generated using R software version 4.0.5.

Figure 6 shows the percentage share of estimated PM_2.5_ cause-specific mortality for 31 NAC. North, Central, East, West and South India has accounted for 31.8%, 7.9%, 13.2%, 29.1% and 18% of total IHD; 27.6%, 15.4%, 14.2%, 28.2% and 14.6% of total COPD; 20.8%, 8.9%, 30.2%, 25.3% and 14.6% of total Stroke; 37.9%, 8.9%, 18.1%, 22.4% and 12.7% of total LC; 26.7%, 14.8%, 14.7%, 29.3% and 14.3% of total LRI; 43.2%, 19.3%, 14.8%, 11.6% and 11.3% of total NCD-other respectively. IHD showed the highest percentage share for most of the NACs ranging from 20 to 72% and LC being the lowest ranging from 1 to 3%. Stroke and COPD almost showed similar variation ranging from 8 to 31% and 8 to 33% respectively. Stroke constituted the highest percentage share at Bhubaneswar and Guwahati. Whereas, for Jaipur and Jodhpur, 33% of the share is constituted of COPD cases. LRI ranged from 7 to 19% and NCD-other from 0 to 25%. Cities like Ludhiana and Amritsar exhibited no NCD-Other cases.

#### Contribution of air pollution to all-cause total mortality

**Figure 7:**
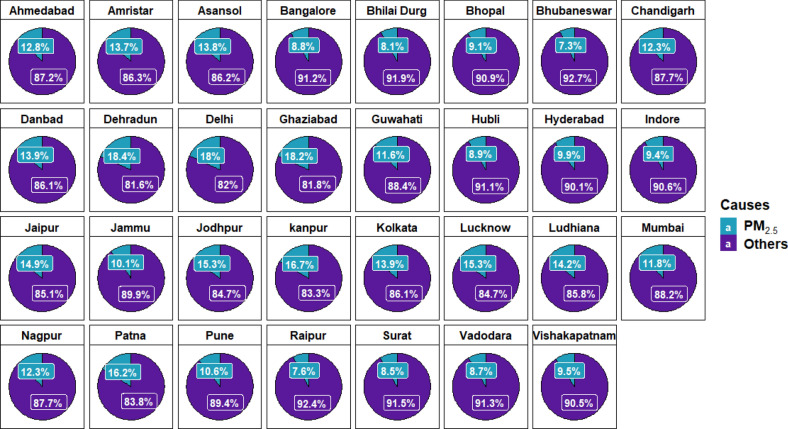
All cause total mortality Vs Estimated PM_2.5_ all cause mortality share (%). The graph wasis generated using R software version 4.0.5.

Figure 7 shows the percentage share of death estimated due to PM_2.5_ pollution. The percentage share of mortality due to PM_2.5_ ranged from 7.2 to 18.4%. For the year 2015^2^ reported that 10.6% of the total premature mortality in the country is attributable to Particulate Matter (PM). The highest range of mortality was observed for North, Central and East zone cities (Table 1). Cities such as Chandigarh (12.3%), Ludhiana (14.2%), Amritsar (13.7%), Dehradun (18.4%), Delhi (18%), Ghaziabad (18.2%), Kanpur(16.7%), Patna (16.2%), Lucknow (15.3%), Kolkata (13.9%) and Asansol (13.8%) showed premature mortality consistently higher irrespective of class as they lie within the IGP region. David et al. (2019) reported that 76% of total premature deaths within the IGP region is due to indigenous emission sources and the rest due to cross-boundary transport and natural resources. He also reported that transboundary movement of pollutants from IGP anthropogenic sources to the North, Central, East, West and South zone accounts for 8% of total premature mortality. Cities like Jaipur (14.9%), Jodhpur (15.3%) in North and ‘X’ class cities like Mumbai (11.8%), Pune (10.6%) and Ahmedabad (12.8%) which showed higher percentage share was due to contribution from anthropogenic sources, outside India and natural sources (David et al. 2019)^64,65^. Central and South Zone cities showed the lowest percentage share of the range 7.6–9.5%. Bangalore being an ‘X’ class city showed a comparatively lower percentage share of 8.8% probably due to low influence from cross-boundary transport of pollutants within and outside Indian regions compared to others (David et al. 2019)^64^ and improved meteorological conditions compared to North India cities^66^. Guttikunda et al.^67^ reported that Central Zone cities like Raipur and Bihali Durg together contributed 17% and 12% of PM_2.5_ from transportation and domestic cooking respectively to ambient air. This is minimal compared to other megacities. Bhubaneswar reported the lowest mortality share of 7.3%. The overall mortality due to air pollution is not just limited to PM_2.5_ concentration but also depend on the population being exposed.

## Monetary estimate of damages due to premature mortality attributable to air pollution

**Figure 8:**
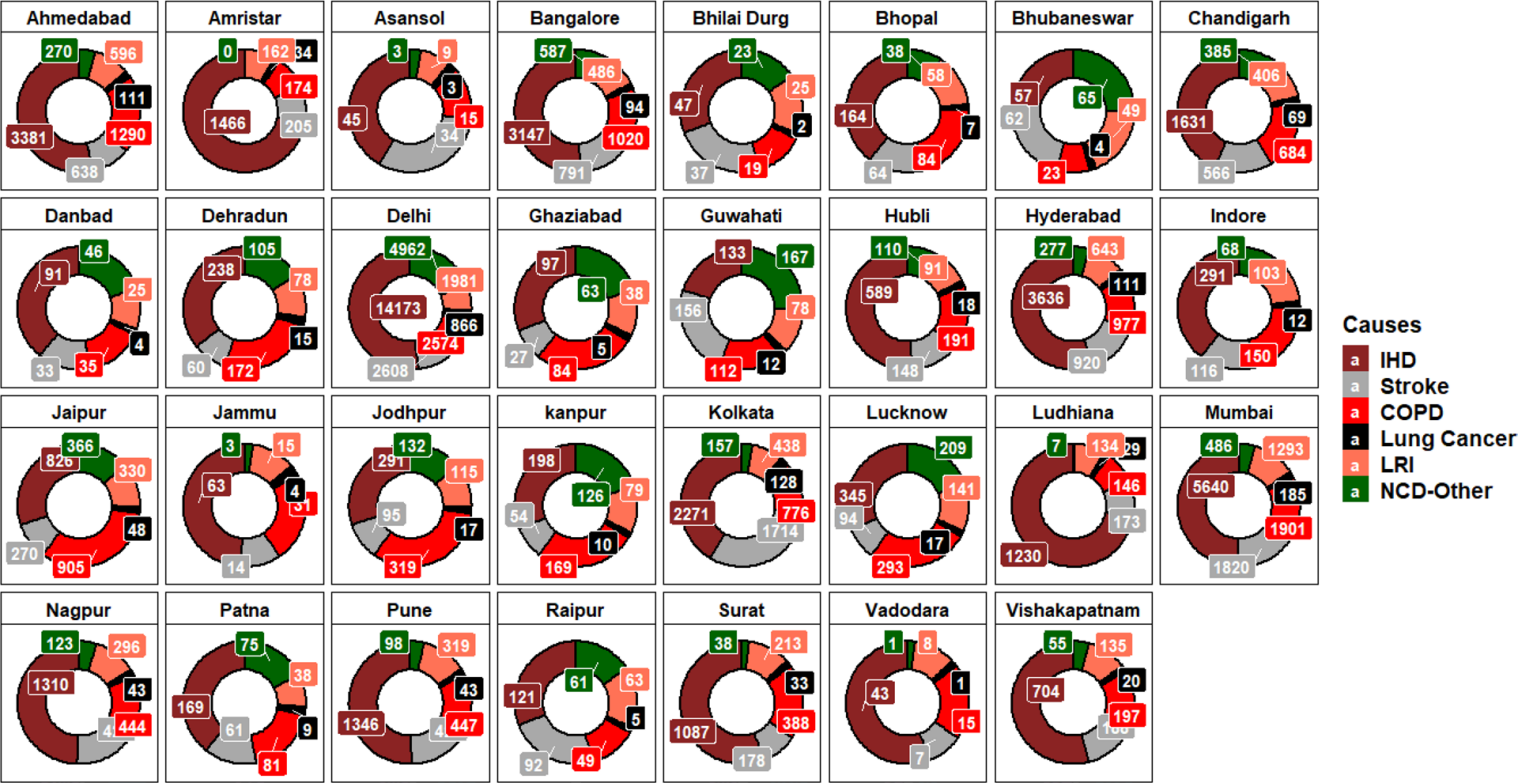
Damage cost associated with estimated PM_2.5_ cause specific mortality (Million US$) for the year 2017. The graph wasis generated using R software version 4.0.5.

**Figure 9:**
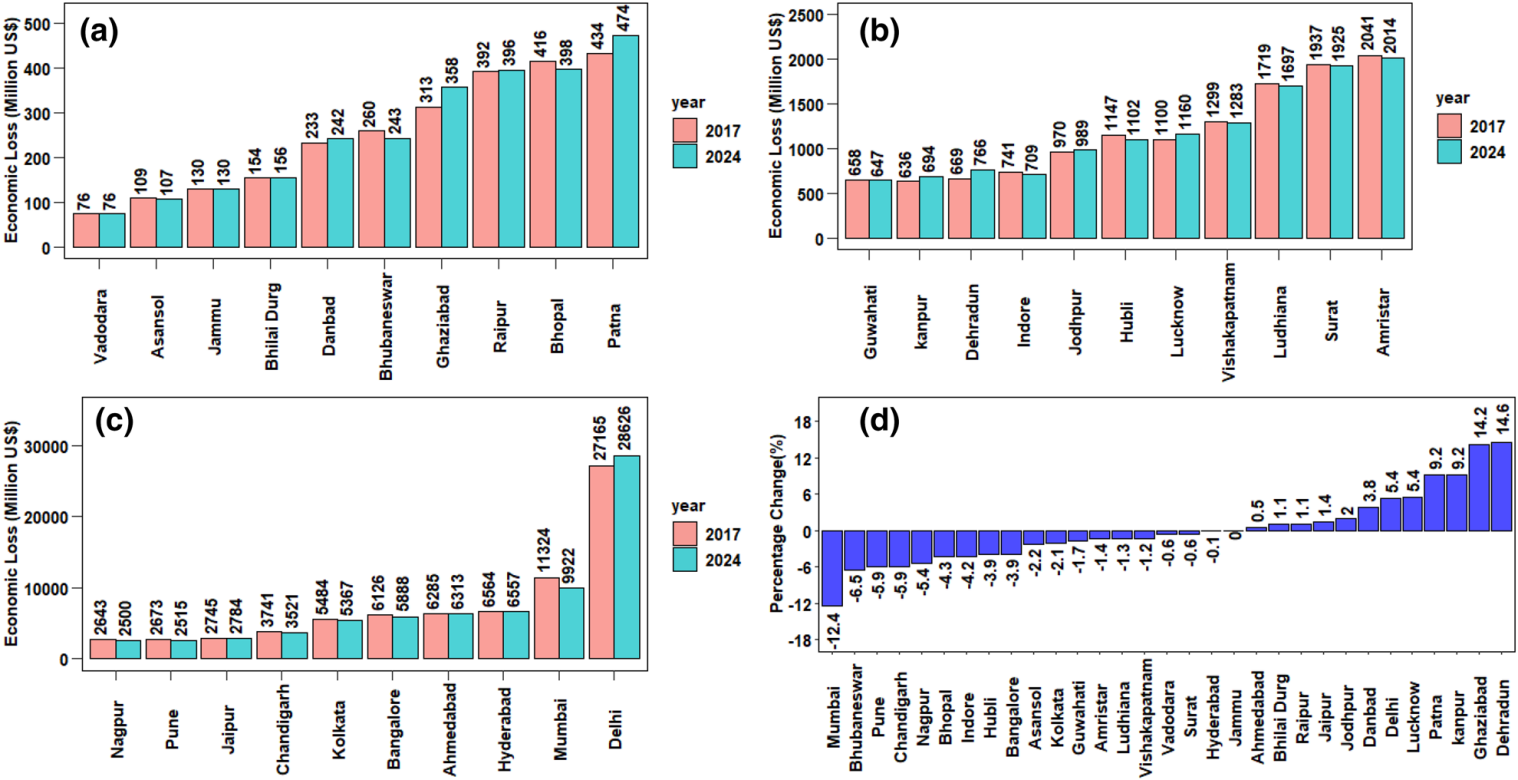
Total Damage cost (Million US$ as of 2017) associated with estimated PM_2.5_ all cause mortality for the year 2017 & 2024 (**a**) < 500, (**b**) >  = 500 & < 2500, (**c**) >  = 2500, d)Percentage change in mortality cases/damage cost based on 30% reduction in PM_2.5_ for the year 2024 from the base year 2017 as per NCAP policy intervention. The graphs are were generated using R software version 4.0.5.

Economic damage (Million US$) associated with estimated cause-specific PM_2.5_ mortality is shown in Fig. 8. PM_2.5_ GEMM (NCD + LRI) mortality for 31 NACs has accounted for total economic damage of 90,185 [95% CI 88,016.4–92,411] million US$ for the year 2017 and is 3.4% of country GDP. The present estimated economic welfare loss is approximately 4 times the total forgone labour output assessed for the year 2019 due to ambient air particulate matter for India by^4^. This is due to the fact that WTP method values all premature mortality due to air pollution identically. Whereas, labour output based method values premature mortality due to air pollution only among the working age groups^68^. Total damage estimated in the study ranged from 76.2 to 27,165 Million US$. The details corresponding to total economic loss attributable to PM_2.5_ mortality for each NACs in the study is depicted in Fig. 9a–c. Of the total damage, 72.8% has resulted from ‘X’ class cities. IHD cause-specific mortality has recorded the highest loss of 44,833 [95% CI 43,819.7–45,871.2] million US$ followed by COPD of 13,768 [95% CI 12,936.9–14,656.2] million US$, Stroke of 12,072.5 [95% CI 11,244–12,966.6] million US$, LRI of 8446.2 [95% CI 7466–9558.7] million US$ and LC of 1958.5 [95% CI 1838.1–2086.4] million US$. Economic damage due to NCD-other was 9106.8 million US$. Highest loss among ‘X’ class cities was recorded for Delhi at 27,165.3 [95% CI 26,403.8–27,948.7] million US$ and lowest for Pune at 2672.8 [95% CI 2611.2–2735.8] million US$. Whereas for ‘Y’ class cities, the highest was for Chandigarh at 3741.4 [95% CI 3651.8–3833.1] million US$ and lowest for Vadodara at 7.6 [95% CI 74.9–77.5] million US$. The total economic loss for Chandigarh was higher despite observing lower GEMM (NCD + LRI) mortality cases compared to other cities like Lucknow, Jaipur, Amritsar, etc. which is due to the higher per capita income of the state. Maji et al.^51^ estimated an average economic loss for Mumbai and Delhi for the year 1995–2015 and reported 1127.2 and 1129.2 million US$ respectively. VSL for India used in his study was computed from US specific VSL reported by^69^ using the benefits transfer approach and is ≈ 24.2% of our value used in the present study resulting in a higher difference in total economic loss. Majumder & Madheshwaran^46^ their study reported that transferring VSL estimated from developed countries to developing countries will yield biased VSL values. In the same study VSL estimated for India from VSL of US using Benefit transfer method resulted in an underestimate. Among the zones, North India was found affected with the highest economic loss of 38,511.7 [95% CI 37,381.2–39,677.0] million US$ and for the rest of the zones is shown in Table 1.

## Scenario setting using existing policy interventions

The set of studies for the year 2017 was repeated for 2024. Policy intervention to reduce the impact of air pollution was found beneficial in reducing total economic loss but was not consistent for all NACs in the study. The inconsistency observed was due to the rise in exposed population (Age > 25) (Supplementary Fig. S2 ranging from 44.1 to 59.9% which does not compensate with the decrease in PM_2.5_^16^. Total economic loss for the year 2024 was estimated to be 89,558.7 [95% CI 87,792.15–91,363.3] million US$. An overall reduction of 0.7% from the base year 2017 was observed for the year 2024. Figure 9a–c shows a potential economic loss (Million US$ as of 2017) for the year 2024 on 31 NACs due to a 30% reduction in PM_2.5_ concentration setting 2017 as the base year. Total Mortality attributable to PM_2.5_ for the year 2024 was estimated to be 79,633 [95% CI 70,228–88,859] which is 0.9% less than that of the base year 2017 and is shown in (Supplementary Fig. S3). GEMM 5-COD was found to capture a higher percentage of GEMM (NCD + LRI) for lower exposure reduction^12^. The estimated PM_2.5_ cause-specific economic loss for the year 2024 is shown in (Supplementary Fig. S4). 50.6% of total economic loss for the year 2024 was attributed to IHD followed by COPD (15%), stroke(12.7%), LRI (9.9%), NCD-others (9.5%) and LC (2.1%). Percentage change in economic loss and total mortality cases are directly proportional ranging from − 12.4 to + 14.6% and is shown in Fig. 9d. Table 1 shows cities located in the West (− 4.1%) and South (− 2.3%) zone showed significant average change compared to that of North (+ 0.03%), East (+ 0.1%) and Central (+ 4.6%) India. All ‘X’ class cities showed significant reduction except Ahmedabad (+ 0.5%) and Delhi (+ 5.4%). Cities majorly residing in IGP states of Delhi, Bihar, and Uttar Pradesh showed the highest economic damage ranging from 5.3 to 14.2% excess from the base year 2017. Dehradun on the other hand also showed 14.6% excess monetary loss in the year 2024. Soni et al.^70^ reported that soil, road dust, industrial activities, transportation activities, and anthropogenic burning to be dominant polluting sources in the Dehradun region along with influence from neighbouring polluted IGP regions. A comparative geographical representation of city-specific economic loss due to estimated PM_2.5_ all-cause premature mortality for the years 2017 and 2024 is shown in Supplementary Figure S5. Maji et al.^16^ carried out scenario modelling for Delhi, Mumbai, Kolkata, Bangalore and reported an increase in mortality by 20% for the year 2024 while implying Best Practise for Emission Control (BPEC) to reduce PM_2.5_ concentration by 45% compared to the base year 2010. He also reported that maximum potential health benefits for all cities will be availed upon reaching IT-3 (15 µg/m^3^) and AQG (10 µg/m^3^) scenarios by 2040. In the current study for the same megacities, a percentage change in mortality by − 2.5% with NCAP policy interventions was observed. This improvement can be largely attributable to higher PM_2.5_ reduction by NCAP policy interventions over BPEC. Higher reduction in mortality can be observed at lower concentration scenarios (such as IT-3, AQG) due to a sharp rise in RR as this function in GEMM follows a supralinear behaviour like IER which flattens at higher PM_2.5_ concentration without resulting in a substantial mortality reduction (Saini and Sharma 2019).

## Study assumptions and limitations

Health risk assessments studies have evolved over the years reducing the uncertainties as these can be the critical factors questioning the viability of any such studies. Several assumptions and limitations are involved in our study are (A) PM_2.5_ concentrations retrieved from ground-based stations are considered as a representative value for the entire city. There may be some degree of misclassification while averaging the PM_2.5_ value due to the spatial dependencies of pollutants^71^. (B) Study do not estimate economic loss associated with PM_2.5_ morbidity, synergic effects, mortality associated with other external events such as accidents due to episodic events like haze. (C) Cause-specific baseline mortality share for each of the NACs used for calculations were specific to their respective state. This is due to the reason that mortality share details were unavailable at city levels. The verbatim share values transferred at the city level irrespective of state may result in underestimating/overestimating premature deaths. (D) The total death reported^35^ can have a certain degree of variation as reported by ^75^. An accuracy test in comparison with Sample Registration Survey (SRS)^72^ value (Detailed in Supplementary Table S5) was carried out. The urban mortality rate was found below the lower limit for Uttar Pradesh and Uttarakhand. The mortality rates reported in^72^ were specific to state-level (Rural and urban class) and not at the district/city level. Any modification in city-level mortality based on values reported in^72^ can escalate the chances of further uncertainty. (E) The change in baseline mortality associated with policy interventions is not taken into account in the current study. The increase in NCD + LRI for some cities for the year 2024 was attributed to increasing urban sprawl, change in lifestyle and ageing^10,16^. (F) Study does not differentiate the associated mortality cases concerning indoor air quality. it is assumed that mortality associated with indoor quality for these ‘X’ and ‘Y’ class cities was minimal due to the improved lifestyle compared to the ‘Z’ class cities of India. (G) In this study, the Chemical composition of PM_2.5_ species with an adverse effect on health^73,74^ was not demarcated and assumes equivalent toxicity same as that of PM_2.5_ mass concentration upon simple addition. (H) Due to paucity in city-specific data, the study assesses the VSL using state-specific GDP and PCI assuming the value to remain uniform for individual states irrespective of the city. However, there exists no agreed method or value for assessing the cost of mortality due to air pollution and are subjective based on the time and level of uncertainty.

## Conclusion

The current study quantifies the economic burden due to premature mortality attributable to PM_2.5_ exposure in 31 NACs for the year 2017. Additionally, the study also quantifies the potential monetary benefits based on target scenarios suggested in National Clean Air Programme(NCAP) for the year 2024. PM_2.5_ attributable total premature mortality cases were estimated to be 80,447, resulting in a total economic loss of about 90,185 million US$ for the year 2017. IHD (47%) contributed the highest mortality followed by Stroke (14.7%), COPD (17.0%), LRI (9.9%) and LC (1.9%). NCD-others which were neglected in previous studies has accounted for 9.3% of total premature mortality resulting in a total economic loss of 9106.8 million US$. 7.6–18.4% of total mortality was attributable to excess PM_2.5_ exposure. The highest PM_2.5_ total premature mortality (Economic burden) of 49,212 (65,621 million US$) and 24,227 (38,511 million US$) was observed for class ‘X’ cities and North India Zone respectively. Despite being subjected to low PM concentration, some major cities reported high mortality cases due to a larger portion of the population being exposed.

A decrease of 0.9% to 79,709 cases and 0.7% to 89,558 million US$ was observed on premature mortality and economic loss respectively for the year 2024. However, it is vital to note that the improvement assessed are limited to 31 of the 122 NACs (ie., 25% of the total NACs). The overall improvement in the air quality of 122 NACs through NCAP is anticipated to facilitate a substantial economic benefit attributable to premature mortality reduction. A reduction by 0.6% to 6.9% in cause-specific mortality cases except for LRI (increase by 5.6%) was observed. The overall improvement observed as a result of policy intervention seems insignificant and needs detailed analysis and insights for improvement. This is probably because no city-specific social indicators such as rising population & baseline mortality rates along with economic indicators such as Per Capita Income & Consumer Price Index were considered in deciding the target criteria, which seems to be the need of the hour. It is clear, that for a country like India with 122 NACs, a policy with immediate effects to alleviate pollution levels in achieving NAAQS was mandatory. Subsequently, there exists a robust requirement of necessitating these previously mentioned indicators in setting target scenarios via policy intervention to ensure maximum health and economic benefits in the identified NAC. It is important to emphasize the India specific cohort studies considering heterogeneous topographic conditions, as the lung capacity and dose–response vary largely subjected to the level of pollution and exposure at which people are inhabited. Such studies can favour in prioritising the cities/district/state that requires stringent abatement rules to improve the well-being of human. It is vital to strengthen the available database such as continuous PM_2.5_ monitoring and subsequent expansion of the monitoring network for improved PM representation, minimising the data gaps in mortality report, Cause-specific mortality reporting at state/district level, Availability of economic indicators at district/city level, VSL survey at state/district/city level. Tuning up these vital requirements can help researchers arrive at an estimate with lower uncertainties benefiting policy makers in decision making. Contemporarily, successful implementation and progress of NCAP actions and targets shall be assessed at ground level for all the NACs to aid the anticipated benefits of improved air quality in the country.

## Supplementary Information

Supplementary Information.

## Data Availability

All data generated or analysed during this study are included in this published article and its Supplementary Information files. The raw datasets and corresponding codes generated during and/or analysed during the current study are available in the Economic-Assessment repository, https://github.com/moorthynair/Economic-Assessment.git.
