# Supplementary material for: Valuing burden of premature mortality attributable to air pollution in major million-plus non-attainment cities of India
Source: Sci Rep. 2021 Dec 2;11:22771. doi: 10.1038/s41598-021-02232-z (PMC8640062; doi:10.1038/s41598-021-02232-z)
Supplement: Supplementary file 1 — Supplementary Information. [file 41598_2021_2232_MOESM1_ESM.docx]

**Supplementary Sheet**

**Valuing burden of premature mortality attributable to air pollution in major million-plus Non-Attainment Cities of India.**

Moorthy Nair^1^, Hemant Bherwani^2,3*^, Shahid Mirza^,2^,Saima Anjum^2^, Rakesh Kumar^2,3^

^1^Asian Development Research Institue (ADRI), Patna (BH), India.

^2^CSIR-National Environmental Engineering Research Institute (NEERI), Nagpur (MH), India

^3^Academy of Scientific & Innovative Research (AcSIR), Ghaziabad, Uttar Pradesh, India

*Corresponding author: Hemant Bherwani, Scientist, CSIR-National Environmental Engineering Research Institute (NEERI), Nagpur, India.

E-mail: h.bherwani@neeri.res.in

**Supplementary Table S1**: List of Non-attainment Cities in the current studywith their respective states and zone

| **Zone** | **States** | **Non-Attainment Cities** |
| --- | --- | --- |
| North India | Chandigarh^*^ | Chandigarh |
|  | Delhi | Delhi |
|  | Jammu & Kashmir | Jammu |
|  | Punjab | Ludhiana |
|  |  | Amritsar |
|  | Rajasthan | Jaipur |
|  |  | Jodhpur |
| Central India | Chhattisgarh | Raipur |
|  |  | BhilaiDurg |
|  | Madhya Pradesh | Bhopal |
|  |  | Indore |
|  | Uttarakhand | Dehradun |
|  | Uttar Pradesh | Lucknow |
|  |  | Kanpur |
|  |  | Ghaziabad |
| East India | Bihar | Patna |
|  | Jharkhand | Danbad |
|  | Odisha | Bhubaneswar |
|  | West Bengal | Kolkata |
|  |  | Asanol |
|  | Assam | Guwahati |
| West India | Gujarat | Ahmadabad |
|  |  | Surat |
|  |  | Vadodra |
|  | Maharashtra | Mumbai |
|  |  | Pune |
|  |  | Nagpur |
| South India | Andhra Pradesh | Vishakhapatnam |
|  | Karnataka | Bangalore |
|  |  | Hubli |
|  | Telangana | Hyderabad |
| **Total = 5** | **Total =19** | **Total =31** |

*Union Territory (UT)

**Supplementary Table S2**: PM_2.5_ data availability from continuous and manual monitoring stations for study regions.

| **Non-Attainment Cities** | **Continuous Monitoring** | **Manual Monitoring** | **Total Monitoring Stations** |
| --- | --- | --- | --- |
| Chandigarh^2^ | 🗴 | 🗸 | 5 |
| Delhi^1,2^ | 🗸 | 🗸 | 23 |
| Jammu^3^ | 🗴 | 🗸 | 3 |
| Ludhiana^1^ | 🗸 | 🗴 | 1 |
| Amritsar^1^ | 🗸 | 🗴 | 1 |
| Jaipur^1^ | 🗸 | 🗴 | 1 |
| Jodhpur^5^ | 🗴 | 🗴 | Nil |
| Raipur^4^ | 🗸 | 🗴 | 2 |
| Bhilai Durg^4^ | 🗸 | 🗴 | 1 |
| Bhopal^2^ | 🗴 | 🗸 | 6 |
| Indore^2^ | 🗴 | 🗸 | 3 |
| Dehradun^5^ | 🗴 | 🗴 | Nil |
| Lucknow^1, 2^ | 🗸 | 🗸 | 3 |
| Kanpur^1^ | 🗸 | 🗴 | 1 |
| Ghaziabad^5^ | 🗴 | 🗴 | Nil |
| Patna^1^ | 🗸 | 🗴 | 1 |
| Danbad^5^ | 🗴 | 🗴 | Nil |
| Bhubaneswar^2^ | 🗴 | 🗸 | 6 |
| Kolkata^2^ | 🗴 | 🗸 | 4 |
| Asanol^2^ | 🗴 | 🗸 | 1 |
| Guwahati^5^ | 🗴 | 🗴 | Nil |
| Ahmadabad^1,2^ | 🗸 | 🗸 | 10 |
| Surat^2^ | 🗴 | 🗸 | 3 |
| Vadodra^2^ | 🗴 | 🗸 | 5 |
| Mumbai^5^ | 🗴 | 🗴 | Nil |
| Pune^1^ | 🗸 | 🗴 | 1 |
| Nagpur^1^ | 🗸 | 🗴 | 1 |
| Vishakhapatnam^1^ | 🗸 | 🗴 | 1 |
| Bangalore^1,2^ | 🗸 | 🗸 | 9 |
| Hubli^2^ | 🗴 | 🗸 | 2 |
| Hyderabad^1,2^ | 🗸 | 🗸 | 13 |

**Data source link:**

^1^<https://app.cpcbccr.com/ccr/#/caaqm-dashboard-all/caaqm-landing>

^2^<https://cpcb.nic.in/displaypdf.php?id=bWFudWFsLW1vbml0b3JpbmcvTG9jYXRpb25fZGF0YV8yMDE3LnBkZg>==

^3^http://jkspcb.nic.in/Content/Amibient.aspx?id=223

^4^https://enviscecb.org/Data/AAQMS/AAQMS.pdf

^5^The cities with no PM_2.5_ moniotring stations. PM_10_ was used as surrogate to derive city specific PM_2.5_ concentrations


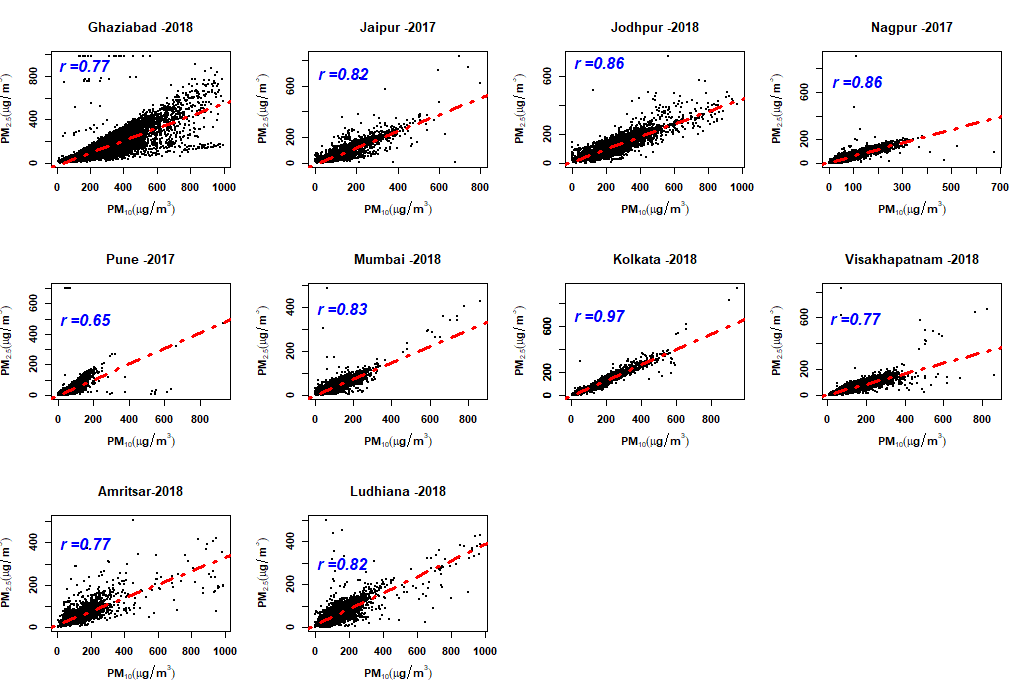


**Supplementary Figure S1:** Pearson correlation between PM_10_ and PM_2.5_ with best fit using linear regression model. The graph is generated using R software version 4.0.5

**Supplementary Table S3:** Best fit equation using linear regression model and previous studies to predict the missing PM_2.5_ concentration using known PM_10_ concentration

| **Cities** | **Best Fit Equation** |
| --- | --- |
| Ghaziabad | PM_2.5_ = -23.6421+0.5727PM_10_ |
| Jaipur | PM_2.5_= -12.5226+0.6468PM_10_ |
| Jodhpur | PM_2.5_ = 6.6315+0.43445PM_10_ |
| Nagpur | PM_2.5_ = 3.3351+0.5671PM_10_ |
| Pune | PM_2.5_ = 0.04597+0.51149PM_10_ |
| Mumbai | PM_2.5_ = -0.3709+0.3707PM_10_ |
| Kolkata | PM_2.5_ = 1.826+0.4147PM_10_ |
| Amritsar | PM_2.5_ = 11.9098+0.3169PM_10_ |
| Ludhiana | PM_2.5_ = 8.618+0.3778PM_10_ |
| Vishakhapatnam | PM_2.5_ = -7.5325+0.6793PM_10_ |
| Kanpur | PM_2.5_ = 0.56PM_10_(CPCB, 2019) |
| Dhanbad | PM_2.5_=0.39PM_10_(Gupta &Elumalai, 2017) |
| Patna | PM_2.5_=0.64PM_10_ (Arif et al., 2018) |
| Dehradun | PM_2.5_=0.59PM_10_(UEPPCB, 2019) |
| Guwahati | PM_2.5_ = 0.55PM_10_ (Tiwari et al., 2017) |


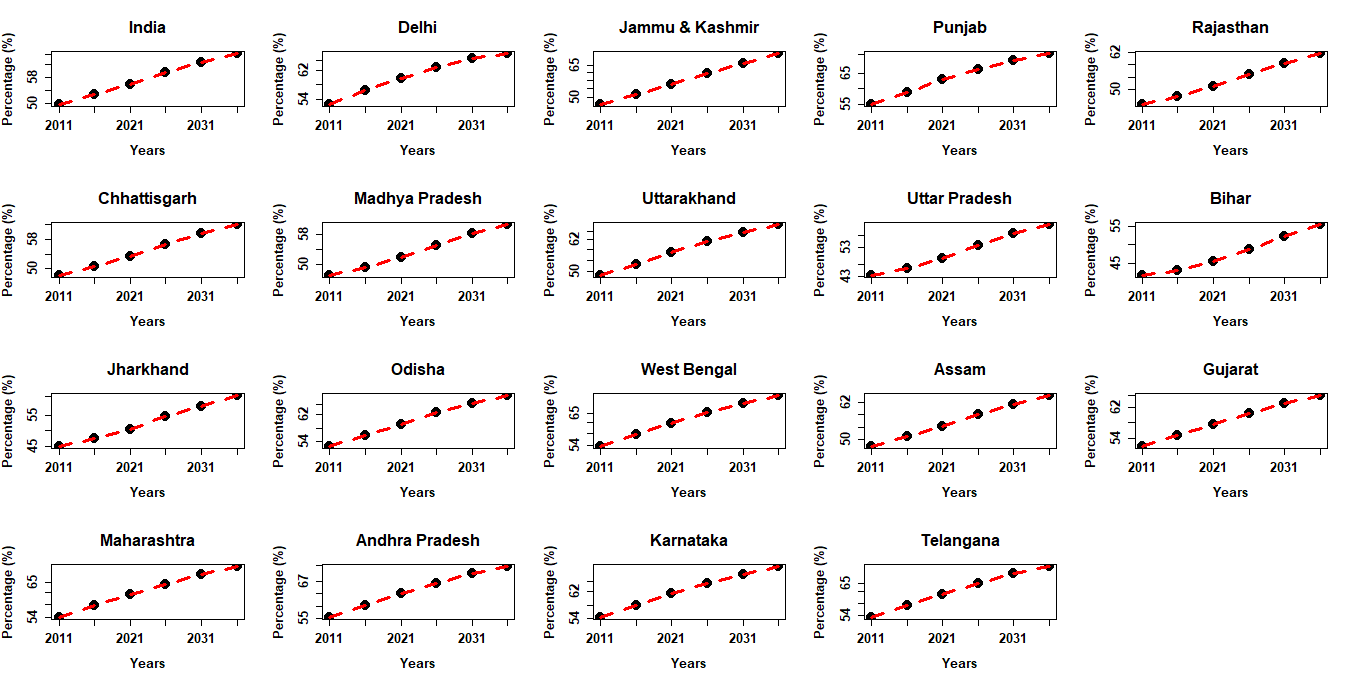


**Supplementary Figure S2:** Percentage share of urban population (Age>25) for each state having NAC in the study from 2011-2036 (CENSUS OF INDIA 2011, 2019). The graph is generated using R software version 4.0.5

**Supplementary Table S4:** Best fit equation using linear regression model for urban population (Age>25) projection for the study years.

| **Cities** | **Best Fit Equation** |
| --- | --- |
| India | Percent (%) = -1217+0.63*Year |
| Delhi | Percent (%) = -1117+0.58*Year |
| Jammu & Kashmir | Percent (%) = -1907+0.97*Year |
| Punjab | Percent (%) = -1285+0.66*Year |
| Rajasthan | Percent (%) = -1307+0.67*Year |
| Chhattisgarh | Percent (%) = -1090+0.56*Year |
| Madhya Pradesh | Percent (%) = -1064+0.55*Year |
| Uttarakhand | Percent (%) = -1492+0.76*Year |
| Uttar Pradesh | Percent (%) = -1415+0.72*Year |
| Bihar | Percent (%) = -1084+0.55*Year |
| Jharkhand | Percent (%) = -1277+0.65*Year |
| Odisha | Percent (%) = -1151+0.59*Year |
| West Bengal | Percent (%) = -1377+0.71*Year |
| Assam | Percent (%) = -1281+0.66*Year |
| Gujarat | Percent (%) = -1020+0.53*Year |
| Maharashtra | Percent (%) = -1235+0.64*Year |
| Andhra Pradesh | Percent (%) = -1243+0.64*Year |
| Karnataka | Percent (%) = -1127+0.58*Year |
| Telangana | Percent (%) = -1365+0.70*Year |

**Supplementary Table S5:**All Cause mortality incidence, Population exposed (Age>25) and Percentage share of GEMM-5COD and NCD+LRI for the year 2017

| **State** | **Non Attainment Cities (NAC)** | **Projected population (age>25)-City level^a^** | **All Cause Mortality –District level(age>25)^b^** | **All Cause Mortality-City level (Age>25)** | **GEMM-5COD^c,d^** | | | | | **NCD+LRI^c,d^** |
| --- | --- | --- | --- | --- | --- | --- | --- | --- | --- | --- |
|  |  |  |  |  | **IHD** | **Stroke** | **COPD** | **Lung Cancer** | **LRI** |  |
| Chandigarh | Chandigarh | 570227 | 16742 | 15684 | 18.1 | 8.5 | 11.3 | 1.0 | 3.6 | 75.6 |
| Delhi | Delhi | 7207637 | 102897 | 69365 | 23.9 | 5.9 | 6.3 | 1.8 | 3.0 | 80.6 |
| Jammu & Kashmir | Jammu | 293458 | 3435 | 2255 | 21.4 | 6.9 | 16.2 | 1.8 | 3.7 | 81.8 |
| Punjab | Ludhiana | 1079291 | 16358 | 12795 | 32.2 | 6.1 | 5.7 | 1.0 | 2.9 | 81.3 |
|  | Amristar | 754949 | 18557 | 15745 | 32.2 | 6.1 | 5.7 | 1.0 | 2.9 | 81.3 |
| Rajasthan | Jaipur | 1686786 | 31666 | 27784 | 12.8 | 5.6 | 20.6 | 0.9 | 4.4 | 75.8 |
|  | Jodhpur | 572433 | 11711 | 9573 | 12.8 | 5.6 | 20.6 | 0.9 | 4.4 | 75.8 |
| Chhattisgarh | Raipur | 615373 | 12759 | 8692 | 11.8 | 14.4 | 7.6 | 0.8 | 4.5 | 72.6 |
|  | BhilaiDurg | 381063 | 6618 | 3223 | 11.8 | 14.4 | 7.6 | 0.8 | 4.5 | 72.6 |
| Madhya Pradesh | Bhopal | 1013363 | 9037 | 8477 | 15.6 | 9.1 | 12.6 | 0.9 | 4.1 | 73.9 |
|  | Indore | 1106836 | 18054 | 14606 | 15.6 | 9.1 | 12.4 | 0.9 | 4.1 | 73.9 |
| Uttarakhand | Dehradun | 359086 | 4982 | 3013 | 15.8 | 5.3 | 16.5 | 1.2 | 4.7 | 77.4 |
| Uttar Pradesh | Lucknow | 1509899 | 20157 | 18685 | 12.6 | 4.6 | 15.7 | 0.8 | 4.6 | 71.2 |
|  | kanpur | 1482158 | 10783 | 9888 | 12.6 | 4.6 | 15.7 | 0.8 | 4.6 | 71.2 |
|  | Ghaziabad | 883632 | 8567 | 4466 | 12.6 | 4.6 | 15.7 | 0.8 | 4.6 | 71.2 |
| Bihar | Patna | 863475 | 15864 | 10625 | 15.6 | 7.5 | 10.9 | 1.1 | 3.2 | 70.2 |
| Jharkhand | Danbad | 647169 | 4965 | 3699 | 15.2 | 7.3 | 8.6 | 0.8 | 3.7 | 69.4 |
| Odisha | Bhubane-shwar | 523448 | 8277 | 6438 | 7.5 | 12.7 | 4.8 | 0.8 | 4.7 | 65.0 |
| West Bengal | Kolkata | 2960580 | 64372 | 64372 | 18.8 | 19.2 | 9.6 | 1.4 | 3.0 | 82.3 |
|  | Asansol | 371278 | 7035 | 1289 | 18.8 | 19.2 | 9.6 | 1.4 | 3.0 | 82.3 |
| Assam | Guwahati | 559201 | 12587 | 11620 | 8.3 | 13.4 | 10.4 | 0.9 | 3.8 | 74.9 |
| Gujarat | Ahmadabad | 3615297 | 46712 | 42975 | 22.8 | 5.8 | 12.9 | 1.0 | 3.3 | 76.7 |
|  | Surat | 2895766 | 21624 | 19923 | 22.8 | 5.8 | 12.9 | 1.0 | 3.3 | 76.7 |
|  | Vadodara | 1082919 | 950 | 769 | 22.8 | 5.8 | 12.9 | 1.0 | 3.3 | 76.7 |
| Maharashtra | Mumbai | 8022599 | 81737 | 81737 | 21.4 | 9.6 | 10.9 | 0.9 | 3.8 | 79.0 |
|  | Pune | 2014590 | 39486 | 21452 | 21.4 | 9.6 | 10.9 | 0.9 | 3.8 | 79.0 |
|  | Nagpur | 1551126 | 24076 | 18221 | 21.4 | 9.6 | 10.9 | 0.9 | 3.8 | 79.0 |
| Andhra Pradesh | Vishaka-patnam | 1204809 | 17624 | 14959 | 21.5 | 8.4 | 9.3 | 0.8 | 3.1 | 74.0 |
| Karnataka | Bangalore | 5619582 | 57647 | 55629 | 21.5 | 8.4 | 11.0 | 0.9 | 2.4 | 78.9 |
|  | Hubli | 628126 | 11530 | 10369 | 21.5 | 8.4 | 11.0 | 0.9 | 2.4 | 78.9 |
| Telangana | Hyderabad | 4545153 | 55487 | 55487 | 21.9 | 7.9 | 9.0 | 0.9 | 2.9 | 73.2 |

^a^(CENSUS OF INDIA 2011, 2019)

^b^(CRS,2017)

^c^(IHME, 2018)

^d^(ICMR, PHFI, and IHME, 2017)


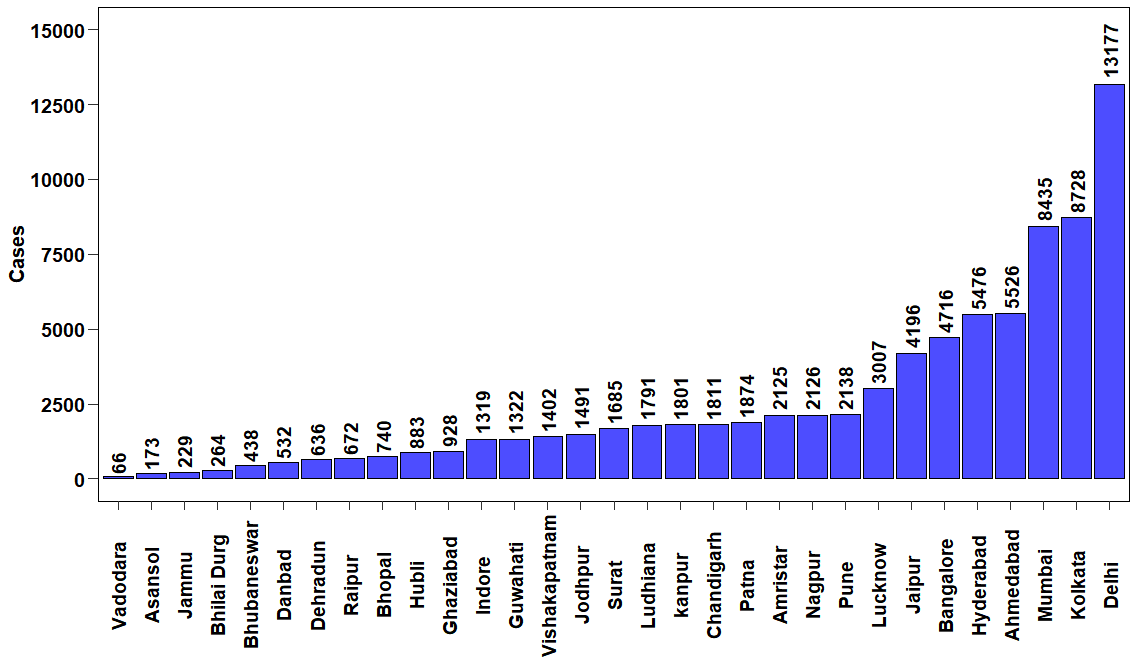


**Supplementary Figure S3:** EstimatedPM_2.5_ all cause mortality cases for the year 2024. The graph is generated using R software version 4.0.5


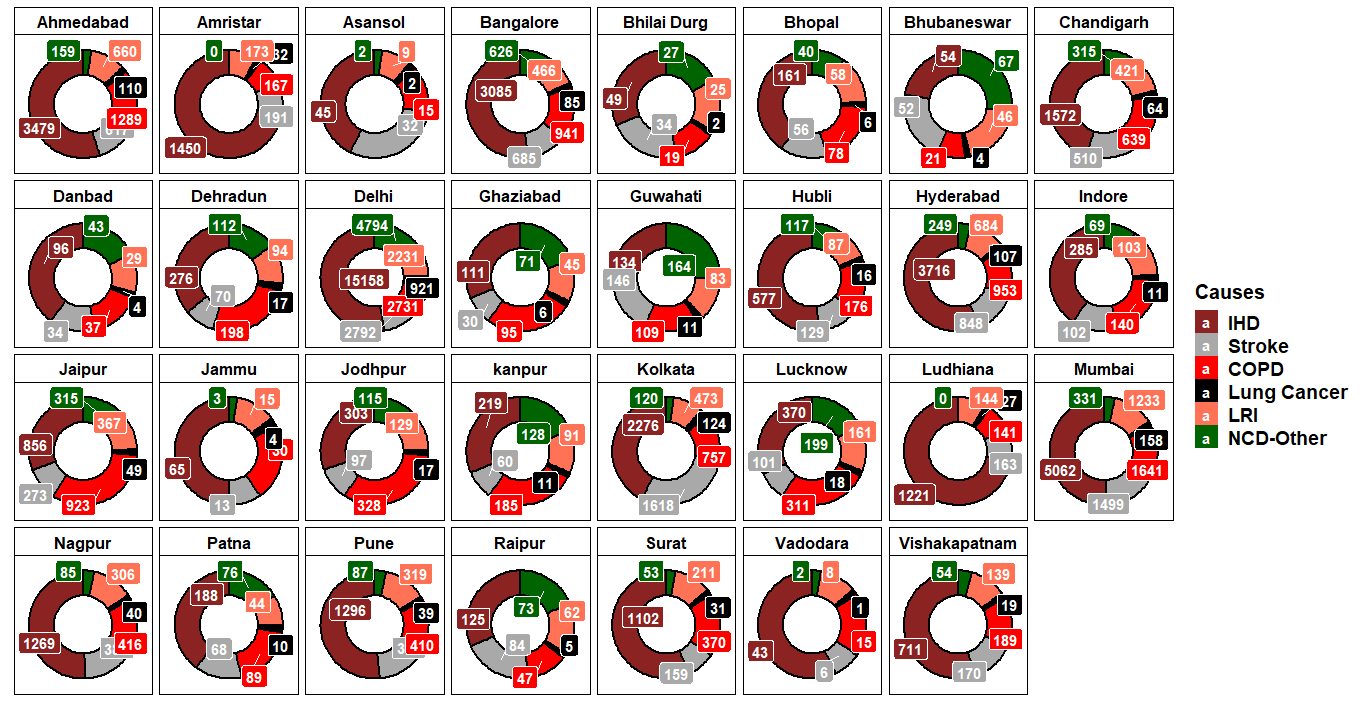


**Supplementary Figure S4:** Damage cost associated with estimated PM_2.5_ cause specific mortality (Million US$) for the year 2024. The graph is generated using R software version 4.0.5


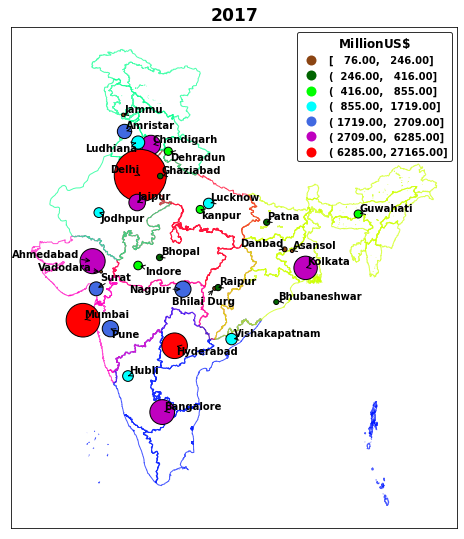

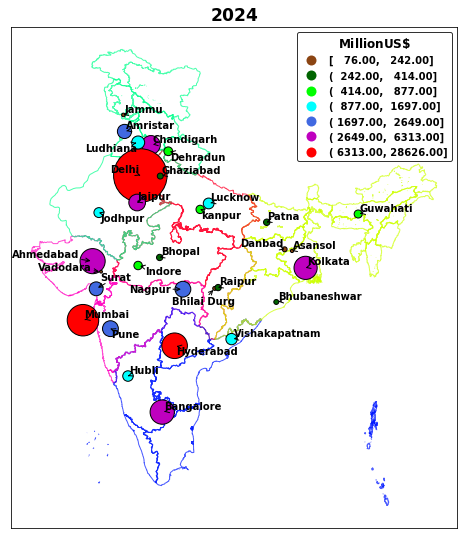


**Supplementary Figure S5:** A comparitive geographical representation of city specific economic loss due to estimated PM_2.5_ all cause premature mortality for the year 2017 and 2024. The maps are generated using Python version 3.8.3.

**Supplementary Table S6:** Validating Civil Registration System (CRS) urban mortality (all ages) with Sample Registration Survey (SRS) values

| **States** | **Death rate.**  **(CRS,2017)** | **SRS report (SRS, 2018)** | | | **ComparativeOutcome** |
| --- | --- | --- | --- | --- | --- |
|  |  | **Death rate** | **Upper Limit** | **Lower Limit** |  |
| Chandigarh | 18.4 | 4.5 | NA | NA | NA |
| Delhi | 6.4 | 3.7 | 4.1 | 3.3 | satisfactory |
| Jammu & Kashmir | 3.8 | 4.3 | 4.7 | 3.8 | satisfactory |
| Punjab | 8.8 | 6 | 6.8 | 5.2 | satisfactory |
| Rajasthan | 7.2 | 5.1 | 5.8 | 4.5 | satisfactory |
| Chattisgarh | 9.2 | 5.6 | 6.5 | 4.7 | satisfactory |
| Madhya Pradesh | 7.3 | 5.5 | 6.2 | 4.9 | satisfactory |
| Uttarkhand | 4.2 | 5.6 | 6.1 | 5.1 | Under reported |
| Uttar Pradesh | 4.4 | 5.4 | 5.9 | 4.9 | Under reported |
| Bihar | 5.1 | 5.4 | 5.7 | 5.1 | satisfactory |
| Jharkhand | 4.3 | 4.6 | 5.1 | 4.1 | satisfactory |
| Odisha | 10.5 | 6 | 6.7 | 5.3 | satisfactory |
| West Bengal | 6.2 | 6 | 6.5 | 5.6 | satisfactory |
| Assam | 7.5 | 5.3 | 5.7 | 4.8 | satisfactory |
| Gujarat | 5.9 | 5.5 | 6 | 4.9 | satisfactory |
| Maharashtra | 6.6 | 4.6 | 4.9 | 4.2 | satisfactory |
| Andhra Pradesh | 8.7 | 5.3 | 6 | 4.5 | satisfactory |
| Karnataka | 7.9 | 4.9 | 5.7 | 4.1 | satisfactory |
| Telangana | 6.2 | 4.5 | 5.1 | 3.8 | satisfactory |

**References**

1. CPCB. (2019). Ambient Air Quality Monitoring Data For The Year 2018. Retrieved from <https://cpcb.nic.in/displaypdf.php?id=bWFudWFsLW1vbml0b3JpbmcvTG9jYXRpb25fZGF0YV8yMDE4LnBkZg==> [Accessed on [Insert Date]
2. Gupta, S. K., &Elumalai, S. P. (2017). Size-segregated particulate matter and its association with respiratory deposition doses among outdoor exercisers in Dhanbad City, India. Journal of the Air & Waste Management Association, 67(10), pp. 1137-1145. https://doi.org/10.1080/10962247.2017.1344159
3. Arif, M., Kumar, R., Kumar, R., Eric, Z., &Gourav, P. (2018). Ambient black carbon, PM2.5 and PM10 at Patna: Influence of anthropogenic emissions and brick kilns. Science of The Total Environment, 624, pp. 1387-1400. https://doi.org/10.1016/j.scitotenv.2017.12.227
4. (UEPPCB), (2019). Report On Action Plan for Air Quality Improvement of Dehradun City. Uttarakhand Environment Protection Pollution Control Board, Dehradun. Retrieved from <http://www.indiaenvironmentportal.org.in/files/file/Air_Action_Plan_of_Dehradun_City_Prepared_by_PCRI.pdf> [Accessed on [Insert date]
5. CENSUS OF INDIA 2011. (2019). Population projections for India and states 2011 – 2036. Report of the technical group on population projections. National Commission on Population Ministry of Health & Family Welfare. Retrieved from <https://nhm.gov.in/New_Updates_2018/Report_Population_Projection_2019.pdf> [Accessed on [Insert date]
6. ICMR, PHFI, and IHME (Indian Council of Medical Research, Public Health Foundation of India, and Institute for Health Metrics and Evaluation). (2017). GBD India Compare Data Visualization. New Delhi. Retrieved [INSERT DATE] from: http://vizhub.healthdata.org/gbd-compare/india
7. IHME (Institute for Health Metrics and Evaluation). (2018). GBD Compare Data Visualization. Seattle, WA: IHME, University of Washington, 2018. Retrieved [INSERT DATE] from: http://vizhub.healthdata.org/gbd-compare
8. SRS. (2019). Sample Registration Survey, Office of the registrar general India, 52(1), https://censusindia.gov.in/vital_statistics/SRS_Bulletins/SRS_Bulletin-Rate-2017-_May_2019.pdf
9. CRS. (2017). Vital Statistics of India based on civil registration system. Office of the Registrar General, India.http://crsorgi.gov.in/web/uploads/download/CRS_report_2017_2020_02_26_revised.pdf
10. Tiwari, S., Dumka, U.C., Gautam, A.S., Kaskaoutis, D.G., Srivastava, A. K., Bisht, D.S., Chakrabarty, R.K., Sumlin, B.J., Solmon, F. (2017). Assessment of PM_2.5_ and PM_10_ over Guwahati in Brahmaputra River Valley: Temporal evolution, source apportionment and meteorological dependence, *Atmospheric Pollution Research*, 8(1), 13-28.
